# CUE domain-containing 2 promotes diffuse large B-cell lymphoma progression via PP2A-B55β degradation and shapes macrophage-mediated immune responses

**DOI:** 10.1186/s43556-026-00626-x

**Published:** 2026-10-13

**Authors:** Li-Jie Zeng, Wei-Xiang Lu, Yuan-Zheng Liang, Jing Yang, Jin-Yan Zhu, Liang Wang

**Affiliations:** Department of Hematology, Beijing Tongren Hospital, Capital Medical University, No. 1 Dongjiaominxiang, Dongcheng District, Beijing, 100730 China

**Keywords:** CUEDC2, PP2A-B55, DLBCL, Ubiquitination, Proliferation, Macrophage polarization

## Abstract

CUE domain-containing 2 (CUEDC2) regulates ubiquitin-mediated protein turnover, cell-cycle progression, and inflammatory signaling, but its role in diffuse large B-cell lymphoma (DLBCL) remains unclear. DLBCL is the most common aggressive non-Hodgkin lymphoma, and a substantial proportion of patients develop relapsed or refractory disease. Here, by integrating clinical bioinformatics, immunohistochemistry, and functional studies in DLBCL cell lines, we demonstrate that CUEDC2 is overexpressed in DLBCL and is associated with advanced disease stage and poor prognosis. Mechanistically, proteomic and ubiquitination analyses revealed that CUEDC2 interacts with the protein phosphatase 2A (PP2A) regulatory subunit B55 and promotes its ubiquitination-associated proteasomal degradation, accompanied by activation of NF-κB and mTOR signaling. Rescue studies support the role of PP2A-B55 as a downstream effector contributing to CUEDC2-associated proliferation and chemoresistance, and isoform-specific experiments identify PP2A-B55β as a preferentially regulated isoform. In addition, CUEDC2 knockdown in macrophages promoted M1-like polarization, enhanced phagocytosis, and supported CD8^+^ T cell activation and lymphoma cell apoptosis in vitro, without a detectable change in PP2A-B55 protein abundance. These immune effects were accompanied by changes in cellular metabolism and histone lactylation, although their causal relationships remain unresolved. Together, these findings support a tumor cell-intrinsic CUEDC2–PP2A-B55β–NF-κB/mTOR axis in DLBCL and suggest a potentially distinct immunomodulatory role for CUEDC2. Further mechanistic studies and in vivo validation are required to establish its therapeutic potential.

**Supplementary Information:**

The online version contains supplementary material available at https://doi.org/10.1186/s43556-026-00626-x.

## Introduction

Diffuse large B-cell lymphoma (DLBCL) is the most common aggressive B-cell lymphoma [1]. Despite advances in treatment, relapsed or refractory disease remains a major cause of poor outcomes [2, 3]. Consequently, identifying novel therapeutic targets for DLBCL remains an unmet clinical need [4].

The CUE domain-containing 2 (CUEDC2) protein is an adaptor protein harboring a ubiquitin-binding motif within a CUE domain [5]. This domain facilitates interactions with monoubiquitin and polyubiquitin chains and regulates substrate stability via ubiquitination [6]. CUEDC2 modulates diverse biological processes, including ubiquitin-mediated protein degradation, cell cycle progression, inflammation, reactive oxygen species production, and tumorigenesis [7–10]. Emerging evidence indicates context-dependent roles for CUEDC2 across malignancies [11–13]; however, its function and mechanisms in DLBCL remain undefined.

Aberrant activation of B-cell receptor (BCR)-related signaling pathways, including NF-κB and PI3K/AKT/mTOR, is a central feature of DLBCL pathogenesis and contributes to tumor-cell survival, proliferation, and therapeutic resistance [14–17]. Protein phosphatase 2 A (PP2A) is a major serine/threonine phosphatase complex that counterbalances phosphorylation-dependent oncogenic signaling. Among its regulatory subunits, the B55 family determines substrate specificity and has been implicated in the control of cell-cycle progression, apoptosis, and kinase signaling [18, 19]. Therefore, dysregulation of PP2A-B55 may provide a mechanism through which ubiquitin-associated protein regulation influences oncogenic signaling in DLBCL.

In addition to tumor-cell-intrinsic signaling, the tumor microenvironment (TME) also plays a critical role in the development and progression of DLBCL. Macrophages and CD8^+^ T cells are key players in shaping the immune microenvironment and influencing tumor progression and behavior [20, 21]. In DLBCL, tumor-associated macrophages (TAMs) are often polarized toward an M2-like phenotype, promoting tumor growth and immune evasion [22, 23]. Similarly, CD8^+^ T cells, which are central to antitumor immunity, are frequently functionally exhausted or dysfunctionally activated within the DLBCL TME, contributing to tumor immune escape [24]. Therefore, whether CUEDC2 participates in regulating immune-cell functions within the DLBCL microenvironment remains an important question.

In this study, we investigated whether CUEDC2 promotes DLBCL progression by destabilizing PP2A-B55β to activate NF-κB/mTOR signaling, and whether it also modulates macrophage-mediated immunity. Using integrated bioinformatics, clinical samples, proteomic and ubiquitination assays, and functional co-culture models, we demonstrate that CUEDC2 is upregulated in DLBCL and is associated with poor prognosis. Mechanistically, CUEDC2 drives ubiquitination-associated degradation of PP2A-B55β, thereby activating NF-κB/mTOR signaling to enhance tumor-cell proliferation and chemoresistance. Additionally, CUEDC2 knockdown in macrophages promotes M1-like polarization and CD8^+^ T cell activation without a detectable change in PP2A-B55 protein abundance.

## Results

### CUEDC2 overexpression correlates with poor prognosis in patients with DLBCL

**Fig. 1:**
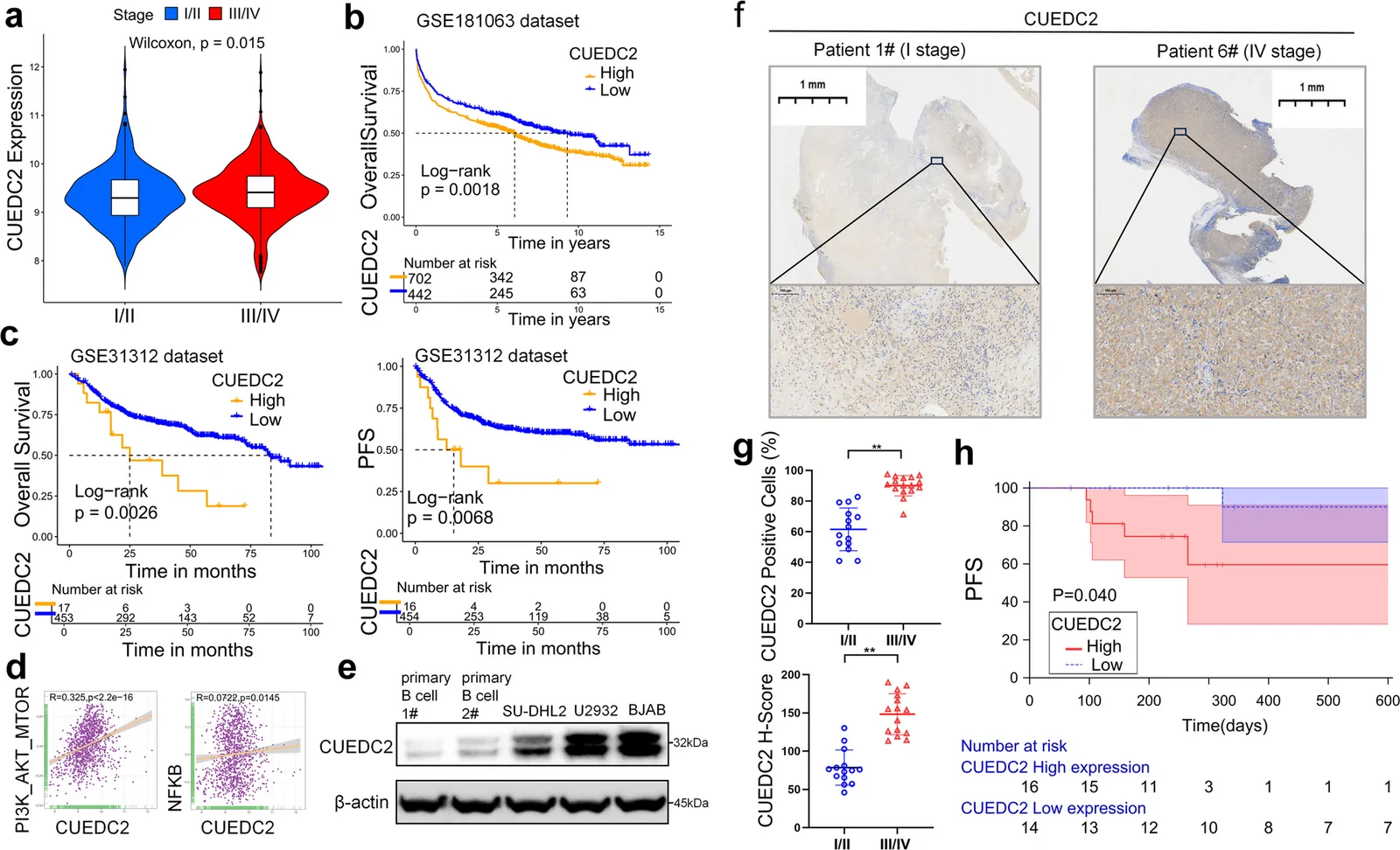
CUEDC2 expression is upregulated in DLBCL and is associated with patient prognosis. **a** CUEDC2 expression in early-stage (I/II; blue) and advanced-stage (III/IV; red) DLBCL in GSE181063; the indicated *P* value was calculated using the Wilcoxon test. **b** Overall survival (OS) in GSE181063 according to CUEDC2 expression. **c** OS and progression-free survival (PFS) in GSE31312 according to CUEDC2 expression. Orange and blue curves indicate high and low expression, respectively; log-rank *P* values are shown. **d** Correlations of CUEDC2 expression with PI3K/AKT/mTOR and NF-κB pathway scores. Purple points represent individual samples, and orange lines show fitted trends with shaded uncertainty bands. **e** CUEDC2 protein levels in primary B cells from two donors and in SU-DHL2, U2932, and BJAB cells; β-actin serves as the loading control. **f** Representative CUEDC2 immunohistochemistry in stage I and stage IV DLBCL, with enlarged views of boxed regions. Brown staining indicates CUEDC2 immunoreactivity. Scale bars in the overview images, 1 mm. **g** CUEDC2-positive cell percentages and H-scores in the clinical cohort (30 patients in total); blue circles and red triangles indicate stage I/II and stage III/IV samples, respectively. Data are presented as mean ± SD; **P < 0.01, independent-samples t test. **h** PFS was estimated using the Kaplan–Meier method and compared using the log-rank test, with the *P* value shown in the panel

To evaluate the clinical relevance of CUEDC2 in DLBCL, we first analyzed public transcriptomic cohorts and then validated CUEDC2 protein expression in clinical DLBCL samples. We analyzed gene-expression data from 1,311 patients (GSE181063), which showed that CUEDC2 expression was significantly upregulated in advanced-stage (III/IV) disease compared with early-stage (I/II) disease (Fig. 1a). Using the *surv_cutpoint* function in the R package *survminer* (based on maximally selected rank statistics), we identified the optimal expression cut-off that best discriminated survival outcomes. Kaplan–Meier analysis revealed that compared with the CUEDC2-low group, the CUEDC2-high group had significantly shorter overall survival (OS) (Fig. 1b). To independently validate these prognostic findings, we analyzed a second public cohort (GSE31312) and confirmed that high CUEDC2 expression was significantly associated with shorter progression-free survival (PFS) and OS (Fig. 1c). Furthermore, CUEDC2 expression was positively correlated with NF-κB and PI3K/AKT/mTOR pathway activity scores and proliferation-related gene-set scores (Fig. 1d and Fig. S1). Finally, Western blot analysis confirmed that compared with that in primary B cells, CUEDC2 protein expression in multiple DLBCL cell lines was elevated (Fig. 1e), supporting the use of these models for subsequent mechanistic studies.

We next performed retrospective immunohistochemical (IHC) staining on 30 clinical DLBCL samples from our center. CUEDC2 protein levels were markedly elevated in advanced-stage disease compared with limited-stage disease, as reflected by both increased positive cell proportions and increased H-scores (Fig. 1f, g). Using an optimal cut-off of 80% positive cells (determined by the Youden index), we stratified patients into high- and low-expression groups. Survival analysis revealed that the high-expression group had significantly inferior PFS (median: 484 vs. 924 days; *P* = 0.040) (Fig. 1h). The patient characteristics are summarized in Table S1. Collectively, these clinical and cellular data indicate that CUEDC2 is upregulated in DLBCL and is associated with adverse clinical features and poor prognosis.

### CUEDC2 promotes tumor growth and chemoresistance

**Fig. 2:**
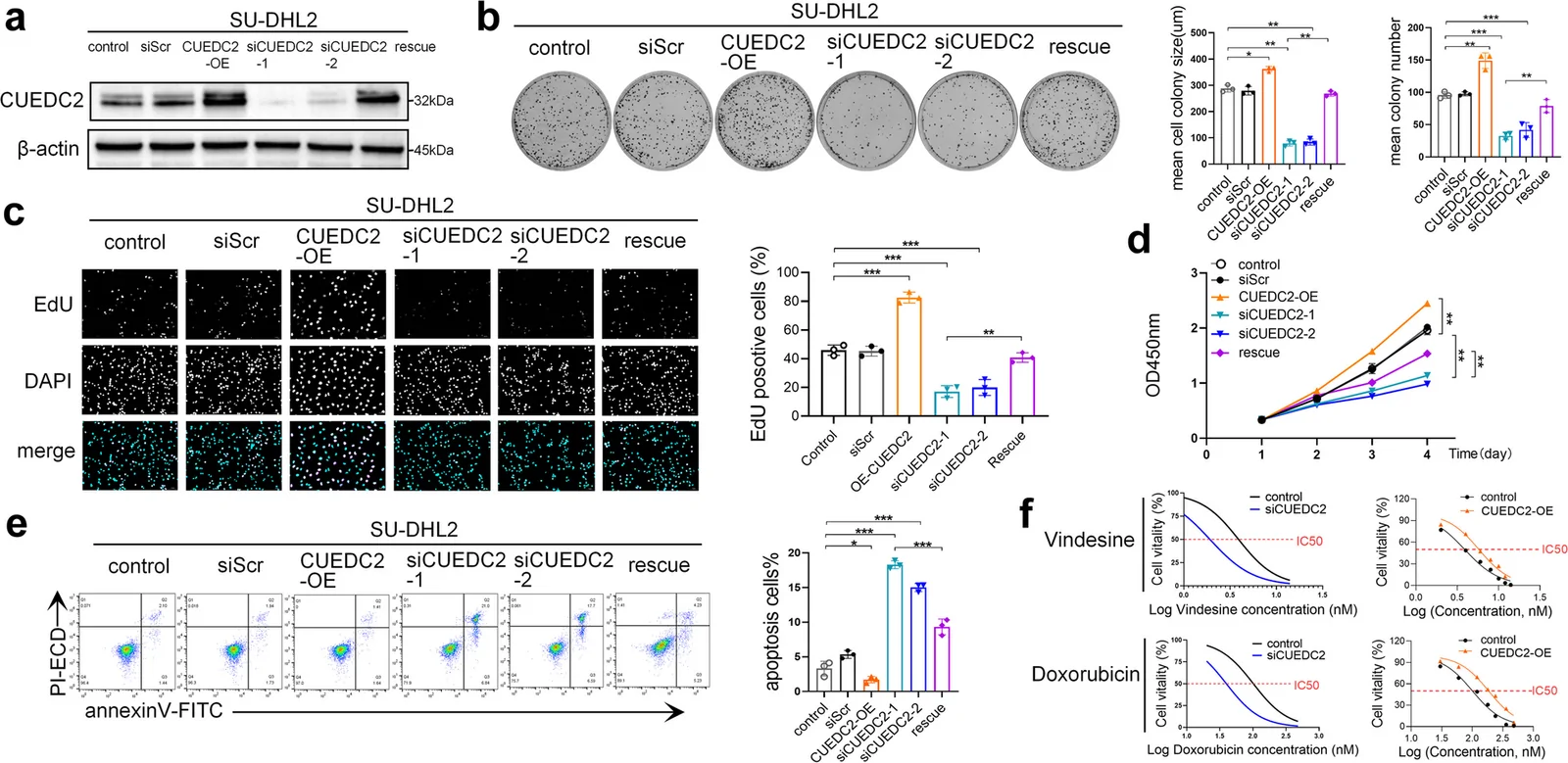
CUEDC2 contributes to DLBCL cell proliferation and chemoresistance. **a** Western blot analysis of CUEDC2 in control, scrambled-siRNA (siScr), CUEDC2-overexpressing (CUEDC2-OE), CUEDC2-knockdown (siCUEDC2-1 and siCUEDC2-2), and rescue SU-DHL2 cells. **b** Soft-agar colony formation and quantification of colony size and number. **c** EdU incorporation and quantification of EdU-positive cells. Individual EdU and DAPI channels are displayed in grayscale; merged images show white EdU signals and cyan nuclear staining. **d** CCK-8 assay of the indicated groups, initially seeded at 2,000 cells per group. **e** Annexin V–FITC/propidium iodide flow cytometry and quantification of apoptosis. **f** Viability after 48 h of vindesine or doxorubicin treatment in control, CUEDC2-knockdown, or CUEDC2-overexpressing SU-DHL2 cells. Red dashed lines indicate 50% viability for estimating half-maximal inhibitory concentrations (IC50). Quantitative data are presented as mean ± SD from triplicate experiments (*n* = 3). *P < 0.05; **P < 0.01; ***P < 0.001, one-way ANOVA; brackets indicate the compared groups

To elucidate the functional role of CUEDC2 in DLBCL, we modulated its expression in SU-DHL2 cells and confirmed the resulting changes in CUEDC2 protein levels (Fig. 2a). CUEDC2 knockdown substantially decreased colony formation, whereas overexpression increased it and re-expression rescued the knockdown phenotype (Fig. 2b). Moreover, cells with suppressed CUEDC2 exhibited a marked reduction in EdU incorporation (Fig. 2c), indicating compromised proliferative capacity, as corroborated by the CCK-8 assay results (Fig. 2d). Consistent with these observations, the percentage of apoptotic cells was significantly greater in CUEDC2-depleted cells, as quantified by flow cytometry (Fig. 2e). We next assessed the response to vindesine, a vinca alkaloid chemotherapeutic agent, and doxorubicin, an anthracycline chemotherapeutic agent. Compared with the control, CUEDC2 knockdown significantly enhanced SU-DHL2 chemosensitivity. The IC50 value for vindesine (48 h) decreased from 3.900 nM (control) to 1.913 nM (siCUEDC2), and that for doxorubicin (48 h) decreased from 101.5 nM (control) to 39.66 nM (siCUEDC2) (Fig. 2f). CUEDC2 overexpression also reduced sensitivity to these drugs (Fig. 2f). In U2932 cells, changes in CUEDC2 expression were confirmed at the protein and mRNA levels (Fig. S2a), with corresponding effects on colony formation (Fig. S2b), CCK-8 signals (Fig. S2c), EdU incorporation (Fig. S2d), drug sensitivity (Fig. S2e), and apoptosis (Fig. S2f). These findings support the reproducibility of the effects in an additional ABC-DLBCL model. Together, these results show that CUEDC2 supports DLBCL cell proliferation and reduces sensitivity to chemotherapy.

### PP2A-B55 regulatory subunit is a novel CUEDC2 interactor

**Fig. 3:**
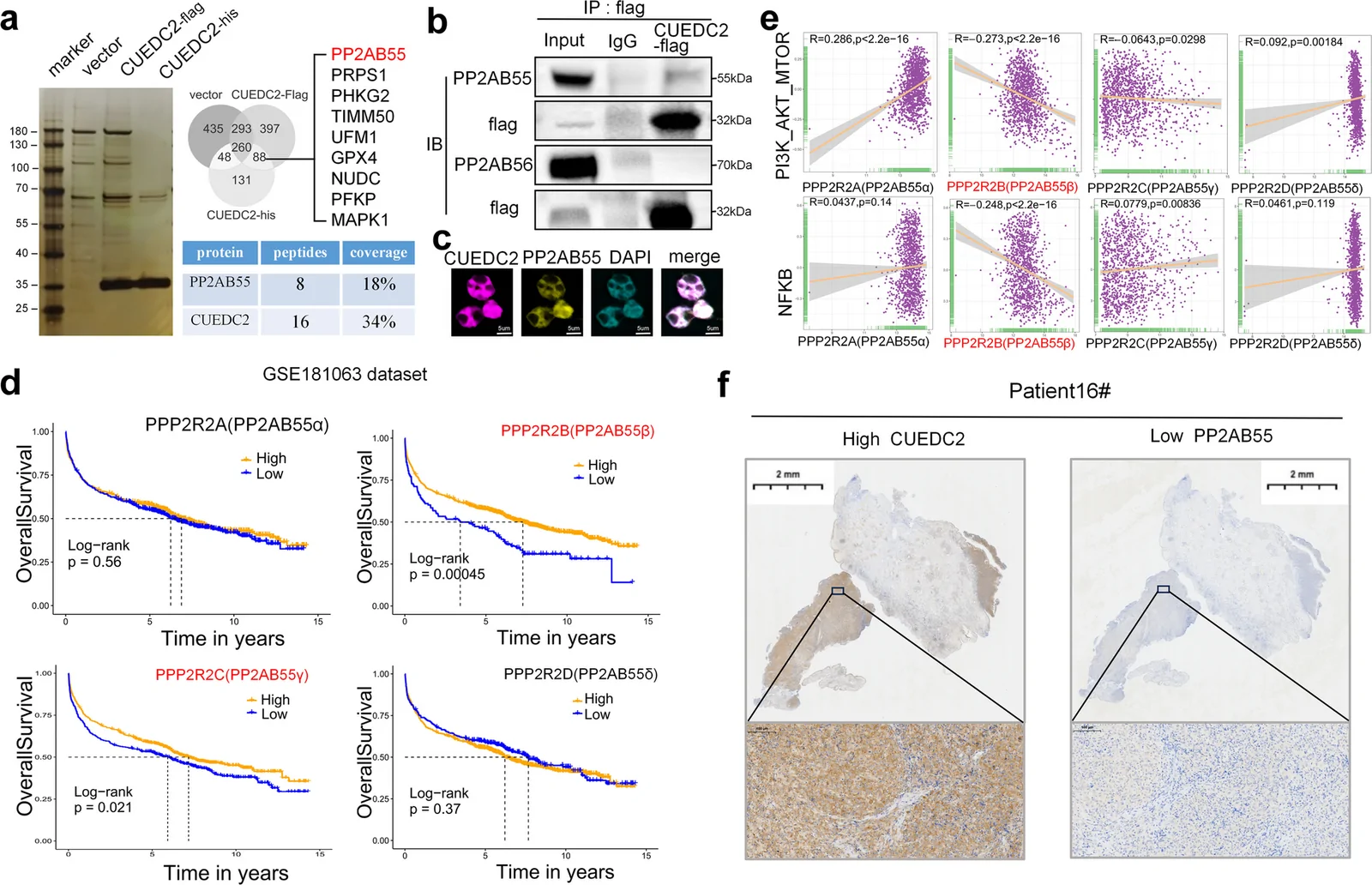
PP2A-B55 associates with CUEDC2 in DLBCL. **a** Immunoaffinity purification and mass spectrometry of CUEDC2-associated proteins in SU-DHL2 cells. The silver-stained gel shows purified proteins; the Venn diagram summarizes overlap between the FLAG- and His-tag purification datasets. The table lists peptide counts and sequence coverage for PP2A-B55 and CUEDC2. **b** Coimmunoprecipitation (Co-IP) of CUEDC2 and PP2A-B55, with PP2A-B56 included as a comparator. **c** Confocal microscopy of CUEDC2 (magenta), PP2A-B55 (yellow), and DAPI-stained nuclei (cyan). **d** OS in GSE181063 according to expression of the B55α, B55β, B55γ, or B55δ isoform; orange and blue curves indicate high and low expression, respectively, and log-rank *P* values are shown. **e** Correlations of B55 isoform expression with PI3K/AKT/mTOR and NF-κB pathway scores; purple points indicate samples, orange lines fitted trends, and gray bands uncertainty around those trends. **f** Representative immunohistochemistry from patient 16, showing high CUEDC2 and low PP2A-B55 staining. Boxed areas are shown at higher magnification. Scale bars in the overview images, 2 mm. IP, immunoprecipitation; IB, immunoblotting

To explore the molecular mechanism by which CUEDC2 promotes DLBCL progression, we performed affinity purification followed by mass spectrometry to identify CUEDC2-associated proteins. Proteomic analysis of FLAG/His-tagged CUEDC2 in SU-DHL2 cells identified 848 potential interactors. After nonspecific binders (e.g., keratins and tubulin) were filtered out, 88 high-confidence interactors remained (Fig. 3a). Co-IP and confocal microscopy further supported the association between CUEDC2 and PP2A-B55 (Fig. 3b, c and Fig. S3a). The B55 regulatory-subunit family comprises four isoforms: B55α, B55β, B55γ, and B55δ, encoded by PPP2R2A, PPP2R2B, PPP2R2C, and PPP2R2D, respectively [18, 19]. Analysis of the GSE181063 cohort revealed that low expression levels of PP2A-B55β and PP2A-B55γ were significantly associated with poor OS (Fig. 3d). Furthermore, PP2A-B55β expression was strongly negatively correlated with cell proliferation and the activity of the NF-κB and PI3K/AKT/mTOR signaling pathways (Fig. 3e and Fig. S3b).

To validate this inverse relationship at the protein level in patient tissues, we performed dual immunohistochemical staining for CUEDC2 and PP2A-B55 on the 30 clinical DLBCL samples described above. We observed that patients with high CUEDC2 expression predominantly exhibited markedly reduced PP2A-B55 staining (Fig. 3f). Correlation analysis showed an inverse trend between the percentages of CUEDC2-positive and PP2A-B55-positive cells, although it did not meet the conventional threshold for statistical significance (*P* = 0.0501; Fig. S4a). We also profiled B55 isoform mRNA expression in BJAB, U2932, and SU-DHL2 cells (Fig. S4b) and compared PP2A-B55 protein levels in these cell lines with those in primary B cells (Fig. S4c). These findings prompted further investigation of the CUEDC2–PP2A-B55 interaction in DLBCL.

### CUEDC2 promotes ubiquitination-associated proteasomal degradation of PP2A-B55β

**Fig. 4:**
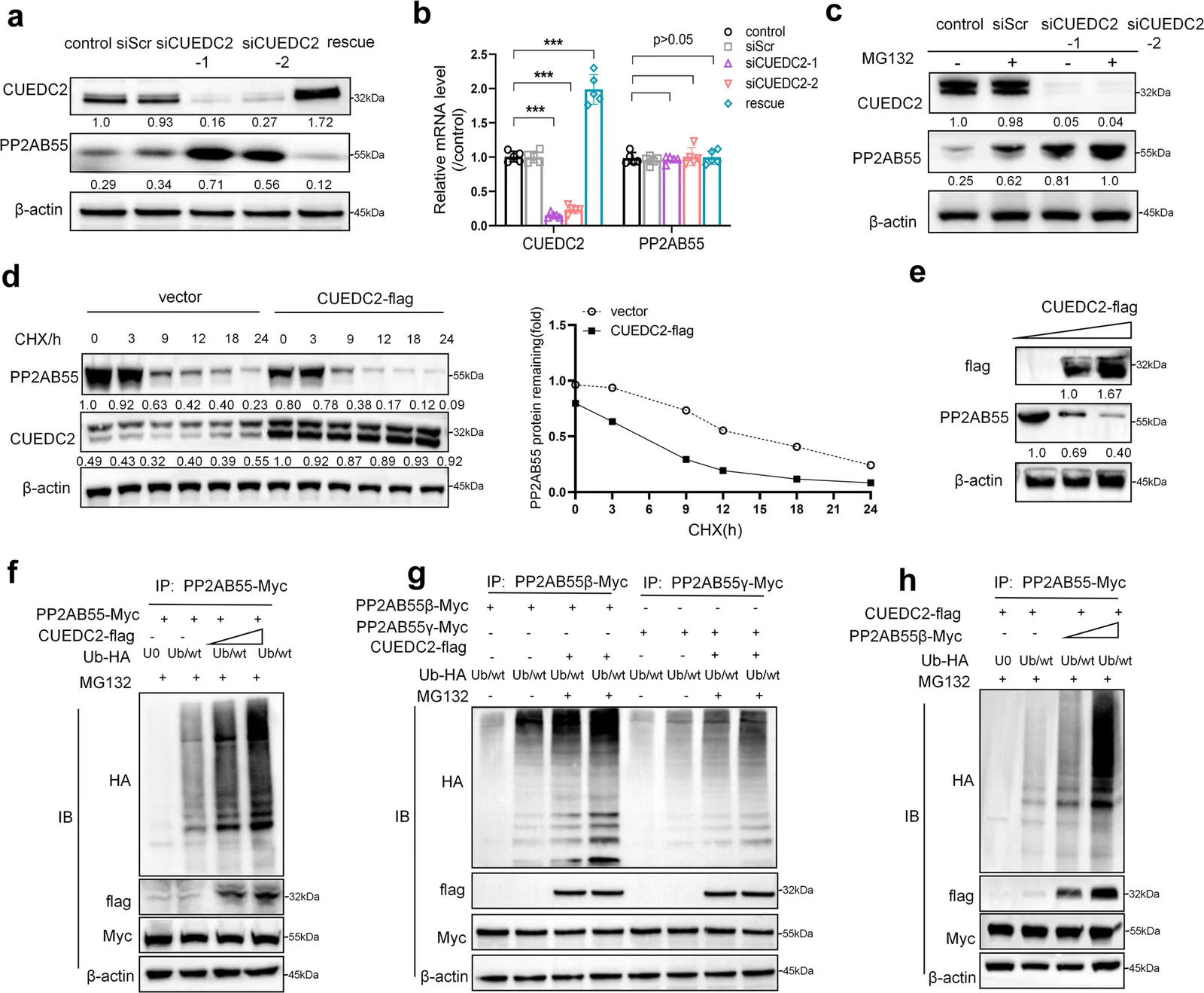
CUEDC2 promotes ubiquitination-associated degradation of PP2A-B55β. **a** Western blot analysis of PP2A-B55 after CUEDC2 knockdown and rescue in SU-DHL2 cells. **b** qRT-PCR analysis of CUEDC2 and PP2A-B55 mRNA. Data are presented as mean ± SD from five biological replicates (*n* = 5); ***P < 0.001, one-way ANOVA. **c** PP2A-B55 protein levels following CUEDC2 knockdown with or without MG132 (10 μM). **d** Cycloheximide (CHX) chase analysis at 0, 3, 9, 12, 18, and 24 h in vector-control and CUEDC2-overexpressing cells. Band intensities were quantified using ImageJ and normalized to the highest value for each measured protein. **e** PP2A-B55 protein abundance with increasing CUEDC2-FLAG expression. **f** Ubiquitination of Myc-tagged PP2A-B55 with increasing CUEDC2-FLAG, HA-tagged wild-type ubiquitin (Ub/wt) or U0 (Ub-K0, a lysine-free ubiquitin mutant in which all seven lysine residues are replaced by arginine), and MG132 as indicated. **g** Comparison of PP2A-B55β and PP2A-B55γ ubiquitination with or without CUEDC2-FLAG and MG132. **h** PP2A-B55β ubiquitination with increasing PP2A-B55β-Myc expression in the presence of CUEDC2-FLAG. Myc-tagged proteins were immunoprecipitated with anti-Myc antibodies and immunoblotted with anti-HA antibodies. Experiments other than (**b**) were performed in triplicate. Input denotes whole-cell lysate; plus and minus signs indicate the presence or absence of the indicated reagent, and wedges indicate increasing construct amounts

CUEDC2 knockdown increased PP2A-B55 protein but not mRNA levels (Fig. 4a, b). Proteasome inhibition (MG132) further increased the PP2A-B55 level (Fig. 4c), suggesting the occurrence of proteasomal degradation. Cycloheximide (CHX) chase assays confirmed that PP2A-B55 protein stability decreased upon CUEDC2 overexpression (Fig. 4d). Increasing CUEDC2 expression reduced PP2A-B55 protein abundance (Fig. 4e). In SU-DHL2 cells expressing Myc-tagged PP2A-B55, anti-Myc immunoprecipitation followed by anti-HA immunoblotting showed increased ubiquitination in the presence of CUEDC2 and HA-tagged wild-type ubiquitin (Fig. 4f). Isoform-specific experiments showed a greater CUEDC2-associated increase in the ubiquitination of PP2A-B55β than PP2A-B55γ (Fig. 4g). An additional assay examined PP2A-B55β ubiquitination with increasing amounts of PP2A-B55β-Myc in the presence of CUEDC2-FLAG (Fig. 4h). Together, these results support preferential regulation of PP2A-B55β by CUEDC2.

### PP2A-B55 regulatory-subunit knockdown promotes tumor growth and chemoresistance

**Fig. 5:**
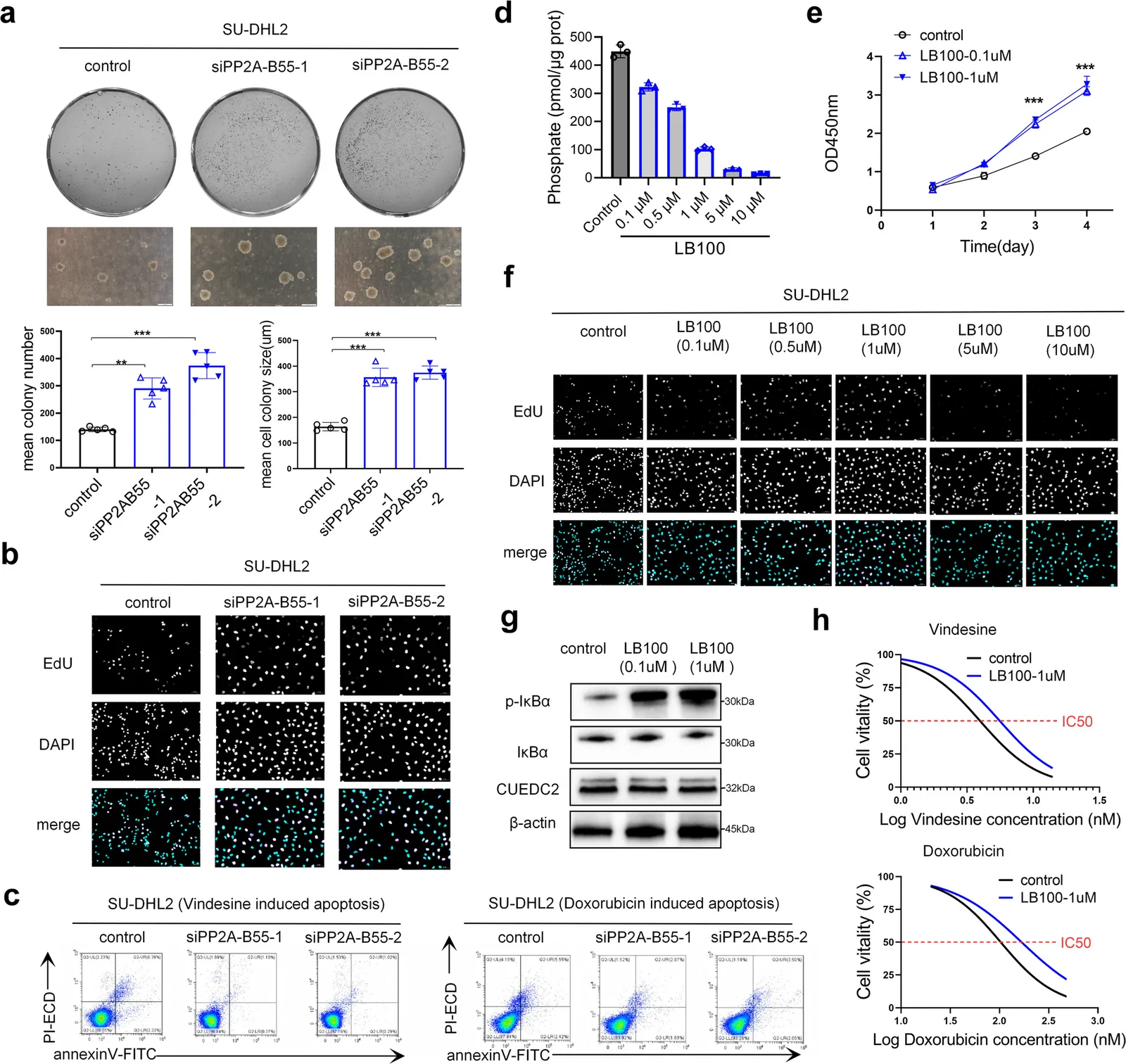
PP2A-B55 knockdown promotes DLBCL cell growth and chemoresistance. **a** Soft-agar colony formation and quantification of colony size and number in control and PP2A-B55-knockdown SU-DHL2 cells. **b** EdU incorporation after PP2A-B55 knockdown. **c** Annexin V/propidium iodide flow cytometry following vindesine or doxorubicin treatment. **d** PP2A activity after treatment with 0.1, 0.5, 1, 5, or 10 μM LB100, measured using the Promega Serine/Threonine Phosphatase Assay System. **e** CCK-8 assay following treatment with 0.1 or 1 μM LB100. **f** EdU incorporation following treatment with the indicated LB100 concentrations. **g** Western blot analysis of NF-κB pathway proteins following treatment with 0.1 or 1 μM LB100. **h** Vindesine and doxorubicin dose–response curves in control cells and cells treated with 1 μM LB100; dashed red lines indicate 50% viability. In EdU images, individual channels are shown in grayscale and merged images show white EdU signals with cyan DAPI-stained nuclei. Quantitative data are presented as mean ± SD from five replicate experiments (*n* = 5). *P < 0.05; **P < 0.01; ***P < 0.001, one-way ANOVA; brackets indicate the compared groups

To test whether loss of PP2A-B55 function phenocopies CUEDC2 overexpression, we knocked down total PP2A-B55 regulatory-subunit expression and pharmacologically inhibited PP2A activity using LB100, a general PP2A catalytic inhibitor. Treatment with siPP2A-B55 significantly increased the colony-forming ability and proliferative capacity of DLBCL cells in vitro, as demonstrated in Fig. 5a, b. More importantly, flow cytometry results showed that the fraction of apoptotic cells induced by vindesine or doxorubicin was significantly reduced in siPP2A-B55-treated cells, as shown in Fig. 5c. Furthermore, treatment with LB100 caused a dose-dependent reduction in PP2A activity: approximately 30% inhibition at 0.1 μM, 45% at 0.5 μM, and 75% at 1 μM (Fig. 5d). At 0.1 μM and 1 μM, LB100 increased CCK-8 signals in SU-DHL2 cells (Fig. 5e). EdU assays showed increased DNA synthesis at lower concentrations but reduced proliferation at 5 μM and 10 μM (Fig. 5f). Low-dose LB100 also increased IκBα phosphorylation (Fig. 5g). Therefore, we chose 1 μM LB100 to treat the cells in subsequent experiments. Compared with the control treatment, the LB100 treatment substantially enhanced SU-DHL2 chemoresistance. Specifically, the IC50 values for vindesine increased from 3.968 nM (control) to 5.592 nM (LB100-1 μM), whereas those for doxorubicin increased from 102.9 nM (control) to 167.8 nM (LB100-1 μM) (Fig. 5h).

These findings indicate that reduced PP2A-B55/PP2A activity promotes DLBCL proliferation and chemoresistance, consistent with a tumor-suppressive role of PP2A-B55 in this context.

### The CUEDC2–PP2A-B55β axis drives DLBCL cell proliferation via the NF-κB and mTOR signaling pathways

**Fig. 6:**
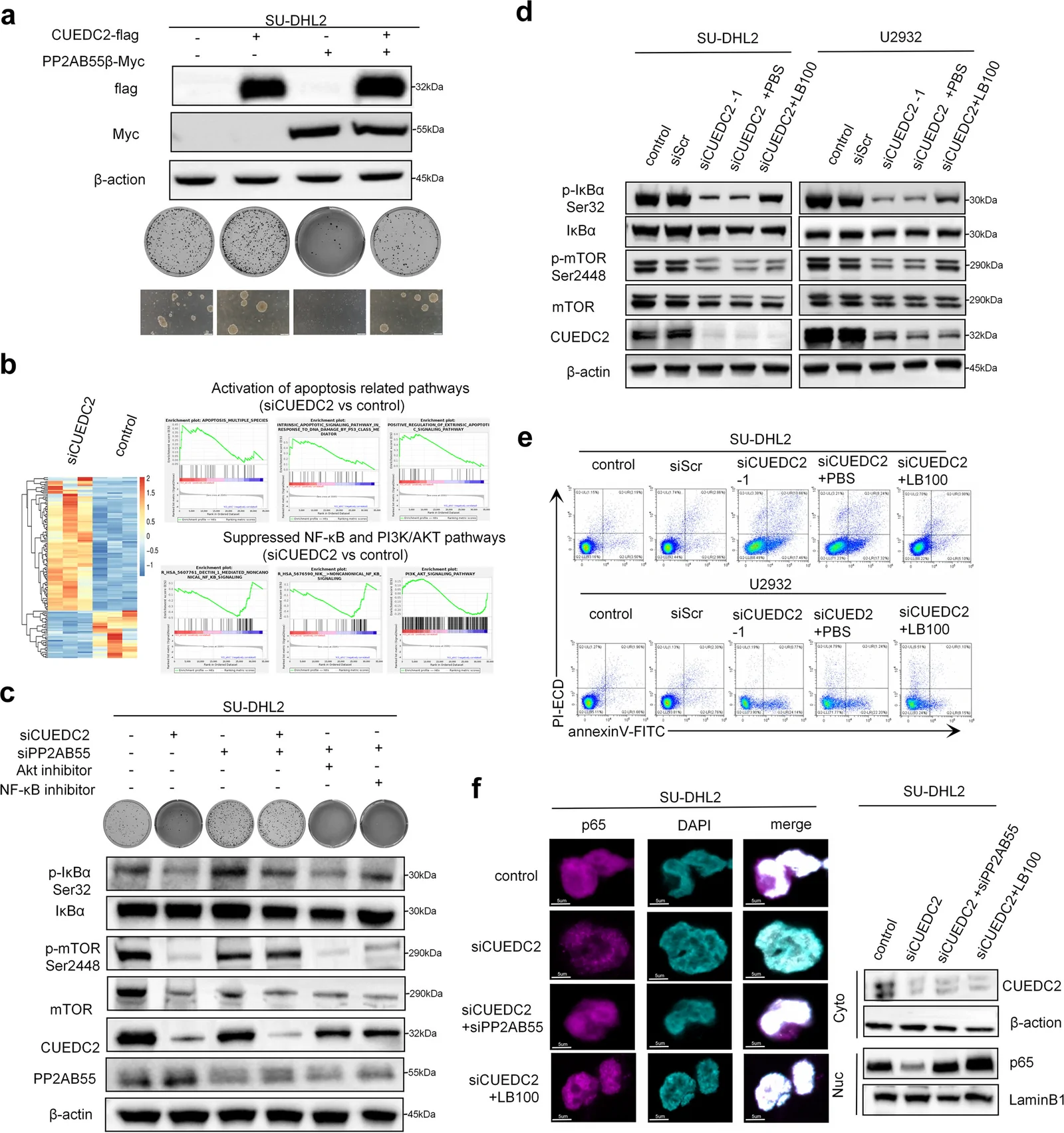
The CUEDC2–PP2A-B55β axis supports DLBCL cell proliferation through NF-κB and mTOR signaling. **a** Western blot and colony formation analyses of CUEDC2-overexpressing SU-DHL2 cells cotransfected with PP2A-B55β. **b** Heatmap of differentially expressed transcripts in control and CUEDC2-knockdown SU-DHL2 cells (1,113 upregulated and 901 downregulated), with GSEA plots for apoptosis, NF-κB, and PI3K/AKT-related gene sets. The heatmap color key indicates relative expression. **c** Colony formation and Western blot analyses following CUEDC2 knockdown, PP2A-B55 knockdown, and treatment with AKT or NF-κB inhibitors in the indicated combinations. **d** NF-κB and mTOR pathway proteins in control and CUEDC2-knockdown SU-DHL2 and U2932 cells treated with or without LB100. **e** Flow cytometry analysis of apoptosis in the corresponding groups. **f** Confocal microscopy of p65 (magenta) and DAPI-stained nuclei (cyan), with cytoplasmic CUEDC2/β-actin and nuclear p65/Lamin B1 immunoblots. PP2A-B55 knockdown restored p65 nuclear localization in CUEDC2-depleted cells. Experiments were performed in triplicate. Plus and minus signs indicate the presence or absence of the indicated treatment

To functionally validate the role of PP2A-B55β as a key downstream effector, we performed rescue experiments in CUEDC2-overexpressing SU-DHL2 cells by cotransfecting PP2A-B55β. Whereas CUEDC2 overexpression alone promoted cell proliferation, the coexpression of PP2A-B55β significantly attenuated this proliferative advantage (Fig. 6a). To delineate the mechanistic role of the CUEDC2–PP2A-B55β axis in DLBCL pathogenesis, we performed RNA sequencing (RNA-seq) on control and CUEDC2-knockdown SU-DHL2 cells. CUEDC2 depletion resulted in the dysregulation of 2,014 transcripts (1,113 upregulated; 901 downregulated). Gene set enrichment analysis (GSEA) revealed significant suppression of the NF-κB and PI3K/AKT pathways (*P* < 0.05; Fig. 6b), aligning with the observed phenotypes: CUEDC2-knockdown cells exhibited increased apoptosis, reduced phospho-IκBα (Ser32/36), and decreased phospho-mTOR (Ser2448) and total mTOR protein levels. PP2A-B55 knockdown partially rescued colony formation and pathway phosphorylation in CUEDC2-depleted SU-DHL2 cells, with AKT and NF-κB inhibitor conditions included to examine pathway involvement (Fig. 6c). LB100 partially restored IκBα and mTOR phosphorylation in CUEDC2-depleted SU-DHL2 and U2932 cells (Fig. 6d) and attenuated apoptosis (Fig. 6e). PP2A-B55 knockdown also restored p65 nuclear localization (Fig. 6f). These rescue experiments support a role for PP2A-B55 in CUEDC2-associated signaling and cell survival.

To assess pathway specificity, we compared control and CUEDC2-knockdown DLBCL cells after IFN-γ or IL-6 stimulation. CUEDC2 knockdown did not appreciably alter IFN-γ-induced STAT1 phosphorylation (Fig. S4d) or IL-6-induced STAT3 phosphorylation under the tested conditions (Fig. S4e).

Given the decrease in total mTOR protein, we next examined a possible RNA-associated contribution to mTOR regulation. KEGG analysis of CUEDC2 RNA immunoprecipitation sequencing (RIP-seq) identified enrichment of signaling pathways including PI3K/AKT (Fig. S5a). Genome-browser tracks showed CUEDC2-associated enrichment over mTOR transcripts relative to the PIK3CD background locus (Fig. S5b), and RIP-qPCR using two CUEDC2 antibodies confirmed mTOR mRNA enrichment relative to IgG controls (Fig. S5c). After transcription was inhibited with actinomycin D, mTOR mRNA declined more rapidly in CUEDC2-depleted cells (Fig. S5d). These findings suggest that CUEDC2 also contributes to mTOR mRNA stability.

Collectively, these data support a model in which CUEDC2 promotes DLBCL proliferation by promoting ubiquitination-associated proteasomal degradation of PP2A-B55β, thereby enabling the phosphorylation of IκBα and mTOR and the consequent activation of oncogenic NF-κB and mTOR signaling.

### CUEDC2 knockdown promotes M1 macrophage polarization and induces tumor cell apoptosis

**Fig. 7:**
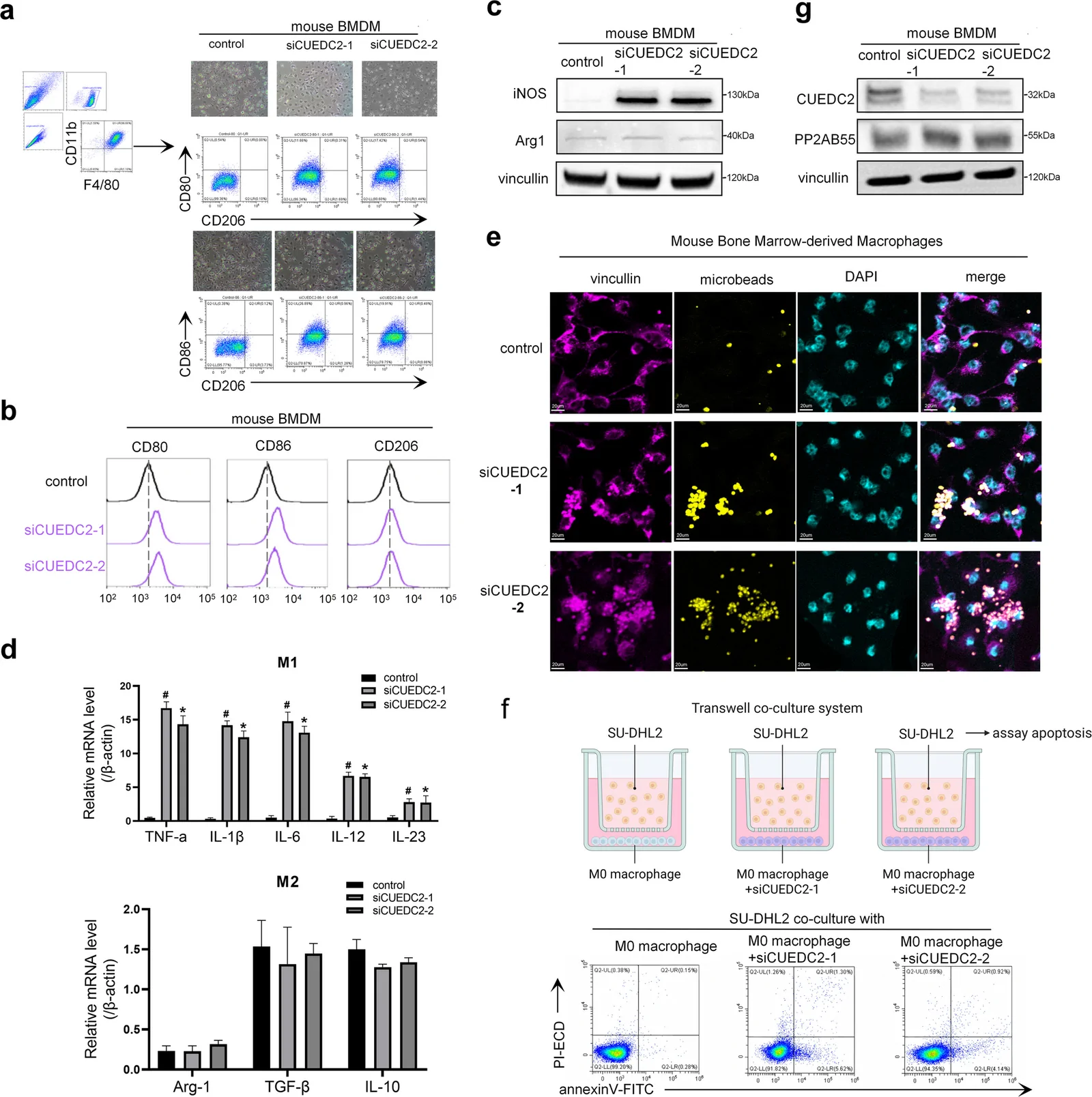
CUEDC2 knockdown promotes M1-like macrophage polarization and lymphoma cell apoptosis. **a** BMDM identification by CD11b/F4/80 flow cytometry, representative morphology, and CD80/CD206 and CD86/CD206 profiles. **b** CD80, CD86, and CD206 expression in control BMDMs and BMDMs transfected with siCUEDC2-1 or siCUEDC2-2; black traces indicate controls and purple traces the knockdown groups. **c** Western blot analysis of iNOS and Arg1, with vinculin as the loading control. **d** Relative mRNA levels of TNF-α, IL-1β, IL-6, IL-12, IL-23, Arg1, TGF-β, and IL-10. **e** Fluorescent bead uptake by BMDMs; pseudocolors show vinculin in magenta, beads in yellow, and DAPI-stained nuclei in cyan. **f** Transwell coculture schematic and apoptosis analysis of SU-DHL2 cells. Orange cells represent SU-DHL2 cells in the insert; blue and purple macrophages represent control and CUEDC2-knockdown BMDMs in the lower compartment, respectively. **g** PP2A-B55 protein levels after CUEDC2 knockdown in BMDMs. Quantitative data are presented as mean ± SD from triplicate experiments. #*P* < 0.05, siCUEDC2-1 versus control; *P < 0.05, siCUEDC2-2 versus control; one-way ANOVA. Schematic in (**f**): Created in BioRender. Zeng, L. (2026) https://BioRender.com/zdcrjzl (publication license agreement no. HC2A95MP7I)

We generated bone marrow-derived macrophages (BMDMs) using a standard protocol to investigate the role of CUEDC2 in macrophage polarization and its effect on tumor cell apoptosis (Fig. S5e). CUEDC2 knockdown in BMDMs increased the expression levels of M1 markers (CD80, CD86, iNOS; Fig. 7a–c), increased the mRNA levels of pro-inflammatory cytokines (TNFα, IL-1β, IL-6, IL-12, and IL-23; Fig. 7d), and enhanced phagocytosis (Fig. 7e). To further explore the role of CUEDC2 in DLBCL progression, BMDMs were treated with control or siCUEDC2. To assess tumor cell apoptosis, SU-DHL2 cells were subsequently cocultured with BMDMs transfected with control siRNA or siCUEDC2. Compared with controls, BMDMs with siCUEDC2 significantly promoted apoptosis in SU-DHL2 cells (Fig. 7f). These findings collectively demonstrate that siCUEDC2 promotes M1 macrophage polarization, which in turn facilitates tumor cell apoptosis. CUEDC2 knockdown did not detectably change PP2A-B55 protein abundance in BMDMs (Fig. 7g), suggesting that the macrophage response may differ from the protein-destabilization mechanism observed in lymphoma cells.

Exploratory analyses further examined the macrophage response to CUEDC2 depletion. GO analysis of CUEDC2-associated RNAs and gene set enrichment analysis identified functional categories related to receptor-mediated endocytosis and phagocytosis (Fig. S6a, b). In metabolic flux assays, CUEDC2-depleted macrophages showed reduced extracellular acidification and a greater oxygen-consumption response to FCCP than control cells (Fig. S6c).

CUEDC2 knockdown was also accompanied by reduced reactive oxygen species signals in macrophages (Fig. S7a). In a separate experiment, BMDMs were exposed to 0–20 mM 2-deoxy-D-glucose (2-DG), and immunoblotting showed changes in protein and histone lactylation, including reduced H3K18 lactylation at higher concentrations (Fig. S7b). CUEDC2 knockdown likewise reduced H3K18 lactylation (Fig. S7c). These exploratory observations link CUEDC2 depletion to metabolic and lactylation changes but do not establish that these changes cause macrophage polarization.

### CUEDC2 knockdown induces M1 macrophage polarization capable of supporting CD8^+^ T cell activation in vitro

**Fig. 8:**
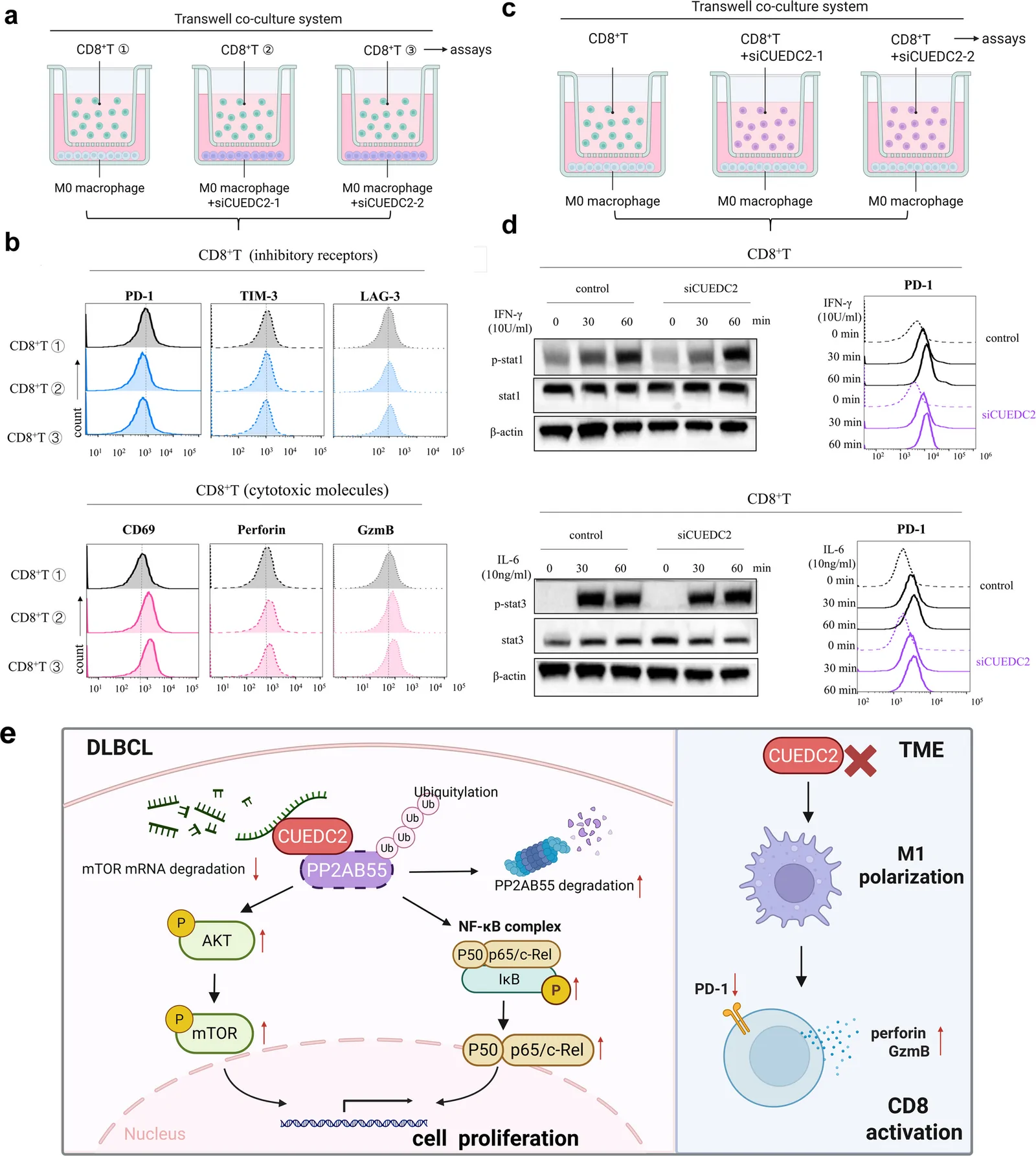
CUEDC2-depleted macrophages support CD8^+^ T cell activation in vitro. **a** Transwell coculture of CD8^+^ T cells with control or CUEDC2-knockdown BMDMs. Teal cells in the insert represent CD8^+^ T cells; blue and purple cells in the lower compartment represent control and CUEDC2-knockdown macrophages, respectively. **b** Flow cytometry profiles of PD-1, TIM-3, LAG-3, CD69, perforin, and granzyme B in the groups numbered in (**a**). **c** Coculture of BMDMs with CD8^+^ T cells directly transfected with control siRNA or siCUEDC2. **d** STAT1 and STAT3 phosphorylation in these CD8^+^ T cells after stimulation with IFN-γ (10 U/mL) or IL-6 (10 ng/mL) for 0, 30, or 60 min, with PD-1 flow cytometry profiles. Black and purple traces indicate control and siCUEDC2 conditions, respectively. **e** Proposed model of CUEDC2-associated tumor-cell signaling and macrophage-mediated immune regulation. Ub denotes ubiquitin and P denotes phosphorylation; the proteasome symbol indicates protein degradation. Upward and downward arrows indicate increases and decreases, respectively, and the red cross denotes CUEDC2 depletion. The macrophage metabolic and histone-lactylation branch represents exploratory associations. Experiments were performed in triplicate. BioRender credits: **a** Created in BioRender. Zeng, L. (2026) https://BioRender.com/7uame97 (publication license agreement no. LT2A95M8MM). **c** Created in BioRender. Zeng, L. (2026) https://BioRender.com/mfyil0m (publication license agreement no. BB2A95MZJR). **e** Created in BioRender. Zeng, L. (2026) https://BioRender.com/s4hprxx (publication license agreement no. KS2A95LWBN)

To determine whether CUEDC2-depleted macrophages influence T-cell activation, we cocultured BMDMs with splenic CD8^+^ T cells and measured immune checkpoint, activation, and cytotoxic effector markers. CUEDC2-depleted BMDMs supported greater CD8^+^ T cell activation, as indicated by decreased expression of the inhibitory immune checkpoint PD-1, upregulated expression of the activation marker CD69, and increased expression of cytotoxic effector molecules, including perforin and granzyme B (Fig. 8a, b). In a separate coculture experiment, CUEDC2 was knocked down directly in CD8^+^ T cells, which were then cocultured with untreated BMDMs (Fig. 8c). This manipulation did not appreciably alter PD-1 expression, IFN-γ-induced STAT1 phosphorylation, or IL-6-induced STAT3 phosphorylation (Fig. 8d). These findings support a macrophage-mediated contribution to CD8^+^ T cell activation in this coculture model. The proposed tumor-cell and immune-cell effects of CUEDC2 are summarized in Fig. 8e.

## Discussion

Our study revealed that CUEDC2 functions as a DLBCL-associated oncogenic factor, with its expression strongly correlated with advanced-stage disease and poor prognosis, a correlation that warrants further exploration in a larger, independent patient cohort. We also revealed a novel role for CUEDC2 in the proliferation of DLBCL cells, expanding upon its established functions in cell cycle regulation, inflammation, macrophage activation, and therapeutic resistance. Mechanistically, we demonstrated that CUEDC2 promotes tumor progression by facilitating ubiquitination-associated destabilization of the tumor suppressor PP2A-B55β, thereby activating the NF-κB and mTOR signaling pathways, which are central to DLBCL pathogenesis. These findings support CUEDC2 as a potential biomarker and candidate therapeutic target; however, further validation is needed before clinical translation.

Protein phosphatase 2 A (PP2A) was initially identified as a tumor suppressor because studies have shown that okadaic acid, a PP2A inhibitor, induces tumors in mice [25]. PP2A is a heterotrimeric complex composed of a catalytic subunit (C subunit), a scaffold subunit (A subunit), and a regulatory subunit (B subunit), the latter providing substrate specificity. Its regulatory subunits fall into four families: B/B55, B′/B56, B″/PR72, and B‴/striatin. The B55 family contains the α, β, γ, and δ isoforms [18, 19]. In this study, we identified PP2A-B55 as a CUEDC2-associated protein in DLBCL cells and found that patients with low expression of PP2A-B55 (PP2A-B55β and PP2A-B55γ) have poor OS.

Currently, PP2A-B55 is known to be involved in the dephosphorylation of key proteins of many signaling pathways, including AKT, Ras, and NF-κB, and in the control of autophagy, metabolism, development, and cell division [26–30]. Moreover, it is modified in cancer at both the genetic and protein levels. A comprehensive study of 995 primary breast tumors revealed both heterozygous and homozygous deletions of the PPP2R2A gene, which correlated with the loss of B55α transcript in patients with ER-positive luminal B breast cancer [31]. Furthermore, somatic deletions were identified in 67% of prostate tumor samples [32]. Genomic deletions affecting B55 regulatory-subunit genes have also been reported in multiple myeloma [18, 33]. In a study of 511 patients with acute myeloid leukemia (AML), B55α protein expression was lower in AML cells than in normal counterparts and was inversely correlated with AKT Thr308 phosphorylation [34]. This observation supports a role for PP2A-B55α in restraining, rather than promoting, AKT phosphorylation in that context.

Our findings support a tumor-suppressive role for PP2A-B55 in the DLBCL models examined here. The increase in IκBα and mTOR phosphorylation following low-dose LB100 treatment is consistent with reduced phosphatase restraint on these pathways. However, PP2A inhibition does not produce a uniform biological response across tumors. Dias et al. showed that PP2A inhibition can hyperactivate oncogenic signaling while inducing cellular stress, creating vulnerability to WEE1 inhibition in colorectal cancer models [35]. This differs from a simple increase in tumor growth and emphasizes the importance of dose, cellular context, and treatment combinations. Moreover, LB100 inhibits PP2A broadly; its rescue effects alone cannot establish B55β-specific activity. Changes in IκBα and mTOR phosphorylation may also reflect indirect effects on other kinases or phosphatases, and direct substrate relationships require biochemical dephosphorylation assays. PP2A-B55 regulation remains incompletely understood [19], and our findings suggest that CUEDC2-dependent protein destabilization is one mechanism contributing to its dysregulation in DLBCL. Conversely, B55α supports oncogenic signaling and malignant properties in pancreatic cancer cells [36]. Together, these observations argue for considering both tumor type and B55 isoform when evaluating therapeutic strategies directed at this phosphatase family.

CUEDC2 has been reported to stabilize SOCS1 and suppress JAK1/STAT3 signaling in AML [8]. JAK/STAT signaling has context-dependent functions in malignant and immune cells [37]. The absence of an appreciable change in cytokine-induced STAT phosphorylation in our DLBCL models suggests that the effect of CUEDC2 cannot be generalized across hematologic malignancies. Our RNA-associated findings also suggest an additional contribution to mTOR regulation beyond PP2A-B55β turnover. However, RIP enrichment demonstrates association with an RNA-containing complex, not necessarily direct RNA binding, and the relationship between this association and transcript stability requires further mechanistic study.

Macrophage states are associated with clinical outcome in DLBCL [38, 39], but their effects depend on cellular context and metabolic state [40]. M2-like programs can favor tumor growth and immune evasion [22, 23, 39], making the macrophage response to CUEDC2 depletion of potential therapeutic interest. Our coculture findings suggest that CUEDC2 targeting may influence both lymphoma cells and macrophage-supported T cell activation. Nevertheless, unchanged PP2A-B55 protein abundance does not establish complete independence from PP2A activity. Nor do the metabolic, ROS, and lactylation observations establish a causal pathway linking CUEDC2 depletion to macrophage polarization. These relationships require targeted rescue and perturbation experiments.

A further consideration is that inflammatory macrophage activation is not uniformly beneficial. Previous work linked CUEDC2 deficiency to macrophage dysfunction and inflammation-associated colon tumorigenesis [41]. Thus, the immune consequences of CUEDC2 inhibition may depend on the tissue environment and duration of treatment. The present in vitro observations support further investigation of macrophage-mediated antitumor responses, but do not yet demonstrate therapeutic efficacy or safety in vivo.

This study has several limitations. First, the findings are primarily based on in vitro models, and in vivo validation is lacking, including in models with an intact immune system. Second, the clinical correlation data, although informative, are retrospective in nature. Third, the detailed molecular mechanism by which CUEDC2 promotes M1 polarization remains to be fully elucidated. Finally, the immunomodulatory effects of CUEDC2 were assessed using mouse-derived immune cells, including coculture with human lymphoma cells; further validation using human primary immune cells is needed.

## Conclusion

CUEDC2 is overexpressed in advanced DLBCL and is associated with poor prognosis. In the tested cell models, it supports lymphoma cell proliferation by promoting ubiquitination-associated proteasomal degradation of PP2A-B55β, which leads to phosphorylation of target proteins and the activation of oncogenic NF-κB and mTOR signaling pathways. Moreover, CUEDC2 knockdown induced M1 macrophage polarization in vitro, supporting CD8⁺ T cell activation and promoting tumor cell apoptosis without a detectable change in macrophage PP2A-B55 protein abundance. These findings support a tumor cell-intrinsic role for the CUEDC2–PP2A-B55β axis in DLBCL progression and suggest a distinct, exploratory immunomodulatory function of CUEDC2 that requires further mechanistic and in vivo validation.

## Materials and methods

### Cell lines and primary B cells

The SU-DHL2, U2932 (ABC-DLBCL subtype), and BJAB cell lines were obtained from the Cell Bank of Shanghai Institute of Cell Biology (Chinese Academy of Medical Sciences, Shanghai, China). All cell lines were authenticated by STR profiling and confirmed to be mycoplasma-free before use. The cells were maintained in Dulbecco’s modified Eagle’s medium (DMEM, Gibco, CA, USA) supplemented with 10% fetal bovine serum (Tico Europe, Netherlands) and 1% penicillin/streptomycin (Gibco, CA, USA) at 37 °C under 5% CO_2_. Cells were treated with serial dilutions of vindesine (0.1–100 nM) or doxorubicin (1–1000 nM) for 48 h before being subjected to CCK-8 or flow cytometry analysis. The primary B cells used as controls were isolated from the peripheral blood of two healthy volunteers from our research group. Peripheral blood mononuclear cells (PBMCs) were isolated by Ficoll-Paque density gradient centrifugation, followed by CD19-positive magnetic bead selection (Miltenyi Biotec).

### Patients

A total of 30 samples from patients with diffuse large B-cell lymphoma (DLBCL) were retrospectively collected from Beijing Tongren Hospital, Capital Medical University. All cases were confirmed by experienced hematopathologists according to the World Health Organization classification of lymphoid neoplasms. The inclusion criteria were as follows: (1) pathologically confirmed DLBCL; (2) sufficient formalin-fixed, paraffin-embedded (FFPE) tissue for immunohistochemical analysis; (3) available essential clinicopathological and follow-up information; and (4) receipt of guideline-recommended standard first-line immunochemotherapy. The exclusion criteria were as follows: (1) transformed or composite lymphoma; (2) primary central nervous system lymphoma or other special lymphoma entities distinct from conventional DLBCL; and (3) relapsed or refractory biopsy specimens from patients already included at initial diagnosis.

### Mouse bone marrow-derived macrophages

Thirty male C57BL/6 J mice aged 6–8 weeks were purchased from Shanghai Model Organisms Center, Inc. (Shanghai, China) and maintained under specific pathogen-free (SPF) barrier conditions. Animal experiments were conducted at Hangzhou Yingchi Biotechnology Co., Ltd. by qualified experimental personnel under an IACUC-approved protocol. Mononuclear cells were isolated from the mouse bone marrow and plated at a density of 1 × 10^7^ cells/mL in a 6-well plate. Macrophage culture medium containing 50 ng/mL M-CSF was added, and cells were cultured for 6 days under standard incubation conditions (37 °C, 5% CO_2_). The medium was changed on Day 3.

### Reagents

IFN-γ and M-CSF were purchased from PeproTech (Rocky Hill, U.S.). LB100 and MG132 were purchased from Topscience (Shanghai, China). Antibodies used in this study are listed in Table S2. CUEDC2-FLAG and PP2A-B55-Myc plasmids were purchased from GENEWIZ (Suzhou, China). In this study, “PP2A-B55” refers to the B55 regulatory-subunit family or total B55 signal detected by the indicated antibody or targeted by the broad B55 knockdown reagent, unless a specific isoform is explicitly stated. “PP2A-B55β” refers specifically to the PPP2R2B-encoded B55β isoform.

### Bioinformatics analysis

Gene set enrichment analysis (GSEA) was conducted using the *c5.all.v7.1symbols.gmt* gene set. A list of gene names and corresponding quantitative values was used as input. The number of permutations was set to 1000. Samples were stratified into control and siCUEDC2 groups, with a false discovery rate (FDR) threshold of < 0.05.

Three DLBCL expression profiling datasets (GSE10846, *n* = 414; GSE181063, *n* = 1,311; and GSE31312, *n* = 498) were retrieved from the Gene Expression Omnibus (GEO) database. Pathway activity scores were calculated using single-sample gene set enrichment analysis (ssGSEA). Hallmark gene sets were used to evaluate NF-κB and PI3K/AKT/mTOR pathway activity. Gene set variation analysis (GSVA) was employed to compute sample-level enrichment scores for five proliferation-related gene sets: G2M_CHECKPOINT, E2F_TARGETS, MYC_TARGETS_V1, PROLIFERATION_SIGNATURE, and CELL_CYCLE. Pearson and Spearman correlation coefficients were calculated between the expression levels of target genes and the corresponding pathway enrichment scores. *P* values were adjusted for multiple comparisons using the Benjamini–Hochberg method.

### Kaplan–Meier plotter analysis

Gene-expression profiles and corresponding clinical data were obtained from GSE181063 (*n* = 1,311) [42, 43] and GSE31312 (*n* = 498). Overall survival and, where available, progression-free survival were analyzed using the Kaplan–Meier method. Expression cut-offs for CUEDC2 and PP2A-B55 isoforms were selected using the surv_cutpoint function in the R package survminer, based on maximally selected rank statistics.

### EdU staining

Cells in the logarithmic growth phase were incubated with EdU solution and subsequently stained using an EdU staining kit (YEASEN BioTECH, Shanghai, China) according to the manufacturer’s protocol.

### Cell Counting Kit-8 (CCK-8) assay

Cells were seeded into 96-well plates in 100 μL of DMEM and cultured for various durations. At the designated time points, 10 μL of CCK-8 reagent (Biosharp, Anhui, China) was added to each well, followed by incubation for 2 h. The absorbance at 450 nm was measured using a microplate reader (Thermo MULTISCAN FC, USA).

### Soft–agar colony formation assay

Cells manipulated through knockdown or treatment with various agents were collected and resuspended as a single-cell suspension in culture medium at a designated concentration (e.g., 1 × 10^6^ cells/mL). The cells were allowed to equilibrate at room temperature, while DMEM containing 10% FBS was prewarmed to 37 °C. Agar was liquefied and held at 56 °C. A bottom layer was created using a mixture of DMEM containing 10% FBS and 0.75% agar, with the top layer comprising 3 × 10^4^ cells in 3 mL of DMEM containing 10% FBS with 0.36% agar. The dish was then incubated at 37 °C for 2 weeks. Colonies were subsequently stained with crystal violet-2% ethanol and imaged.

### Cell flow cytometry

After staining with an apoptosis assay kit (PI, Invitrogen), apoptosis was detected by flow cytometry using a Beckman Coulter instrument. The cells were gently mixed and incubated at room temperature for 15 min. For intracellular flow cytometry, cells were permeabilized with True-Phos™ Perm Buffer (BioLegend) according to the manufacturer’s protocol.

### Western blotting

Whole-cell lysates were collected from treated cells and mixed with 5 × SDS-PAGE loading buffer. After boiling, the proteins were separated by SDS-PAGE and transferred onto polyvinylidene fluoride membranes. The membranes were incubated with primary antibodies and HRP-conjugated secondary antibodies.

### Real-time PCR assays

Total RNA from cells was isolated with TRIzol reagent (Invitrogen), and first‐strand cDNA synthesis was performed using a reverse transcription system (Roche). qPCR was performed using Power SYBR Green PCR Master Mix (Roche) and a LightCycler 480 Sequence assay system (Roche) to quantify all the gene transcripts. Samples were subjected to real-time RT-PCR using appropriate primers (Table S3) for genes of interest, and β-actin was used as an internal control. The fold changes in the target genes normalized to β-actin and the relative expression of the samples were calculated for each sample using the 2^−ΔΔCT^ method.

### Affinity purification of CUEDC2-binding proteins and silver staining

Flag-tagged CUEDC2 transiently expressed in SU-DHL2 cells was purified with anti-FLAG-M2 beads (A2220, Sigma) in Flag-tag protein purification buffer (50 mM HEPES [pH 7.5], 150 mM NaCl, 1 mM dithiothreitol, 0.05% NP-40, and 1 mM phenylmethanesulfonyl fluoride) according to the manufacturer’s instructions. Cellular extracts were subjected to NuPAGE 4%–12% Bis‐Tris gel electrophoresis (Invitrogen). Gel pieces containing protein samples from different labels were excised and digested with trypsin, followed by LC–MS/MS analysis for further characterization. Candidate interactors were selected using the following criteria: (i) identification in both FLAG-CUEDC2 and His-CUEDC2 purifications; (ii) exclusion of common contaminants (keratins, tubulin, and ribosomal proteins); (iii) peptide coverage ≥ 10% and a peptide-spectrum match (PSM) count ≥ 5; and (iv) absence in a control IgG purification.

### Coimmunoprecipitation (Co-IP)

SU-DHL2 cells were seeded in 10-cm dishes, transfected with a Flag-CUEDC2 plasmid, and lysed with IP buffer (20 mM Tris–HCl, pH 7.4; 150 mM NaCl; 1 mM EDTA; 1% NP-40; and 1 × protease and phosphatase inhibitor cocktail). The lysates were rotated for 30 min, after which the proteins were immunoprecipitated using anti-FLAG antibody or IgG in conjunction with prewashed protein A/G magnetic beads (MedChemExpress, Shanghai, China). After overnight incubation at 4 °C, the magnetic beads were washed and eluted in SDS loading buffer, heated at 96 °C for 10 min, and loaded onto a polyacrylamide gel for analysis.

### Cell-based ubiquitination assay

SU-DHL2 cells expressing full‐length PP2A-B55 and HA‐ubiquitin were cultured with varying levels of CUEDC2 expression. Cells were lysed using RIPA buffer (50 mM Tris‐HCl, pH 8.0; 300 mM NaCl; 1% NP-40; 0.5% sodium deoxycholate; 0.1% SDS; and protease inhibitors) at 4 °C for 30 min, followed by centrifugation at 15,000 rpm for 15 min. The protein complexes in the supernatant were subsequently purified using anti‐Myc affinity gel for 1.5 h and then washed five times with RIPA buffer at 4 °C for 5 min each. The eluted proteins were analyzed by immunoblotting using specific antibodies.

### Immunofluorescence assay

Cells were plated, fixed on coverslips, and permeabilized with 0.5% NP-40 for 20 min. Following blocking with 5% BSA for 30 min, the samples were incubated with primary antibodies for 2 h at room temperature. Afterwards, they were probed with fluorescein-conjugated secondary antibodies for 1 h and counterstained with 4′,6-diamidino-2-phenylindole (DAPI). The slides were observed under a confocal microscope (Zeiss, Germany).

### Phagocytosis assay

BMDMs were plated on glass coverslips and incubated with complete medium for 24 h, followed by incubation with control siRNA or siCUEDC2 for another 24 h. Fluorescent latex beads (carboxylate-modified polystyrene, fluorescent red, 0.5-μm mean particle size; Sigma, L3280) were added to the cells at a final concentration of 0.1 μg/mL and incubated for 2 h. After rinsing with Hank's Balanced Salt Solution (HBSS) to remove excess beads, the cells were fixed in 4% paraformaldehyde (PFA) at room temperature for 20 min. Then, the samples were processed according to the immunofluorescence assay protocol.

### RNA interference

All siRNA oligonucleotides were transfected using Lipofectamine RNAiMAX (Invitrogen) as recommended by the manufacturer. The cells were collected 48 or 72 h later, depending on the purpose of each experiment. Control siRNA, CUEDC2 siRNA and PP2A-B55 siRNA were purchased from GenePharma. The target sequences of the individual siRNAs are shown in Table S4.

### Statistical analysis

The data were plotted using GraphPad Prism 9.0 software. To evaluate statistically significant differences, independent-samples t tests were employed for pairwise comparisons. For analyses involving multiple groups, one-way analysis of variance (ANOVA) followed by the Bonferroni correction for multiple comparisons was used. The software package SPSS, version 17.0, was used for all statistical analyses. The results are presented as the mean ± standard deviation (SD), and a *P* value of less than 0.05 was considered to indicate statistical significance.

## Supplementary Information

Supplementary Material 1.

## Data Availability

The raw sequencing data generated in this study have been deposited in the NCBI Sequence Read Archive (SRA) under BioProject accession number PRJNA1462187 (https://www.ncbi.nlm.nih.gov/sra/PRJNA1462187). The data are scheduled for public release on 2027–06-01 and will be accessible via the provided link. All other data supporting the findings of this study are available from the corresponding author upon reasonable requests.
