# Supplementary material for: CUE domain-containing 2 promotes diffuse large B-cell lymphoma progression via PP2A-B55β degradation and shapes macrophage-mediated immune responses
Source: Mol Biomed. 2026 Oct 13;7:208. doi: 10.1186/s43556-026-00626-x (PMC13658750; doi:10.1186/s43556-026-00626-x)
Supplement: Supplementary file 1 [file 43556_2026_626_MOESM1_ESM.pdf]

CUE Domain-Containing 2 Promotes Diffuse Large B-Cell Lymphoma Progression via PP2A-B55β Degradation and Shapes Macrophage-Mediated Immune Responses

Authors: Li-jie Zeng[1†], Wei-xiang Lu[1†], Yuan-zheng Liang[1], Jing Yang[1], Jin-yan Zhu[1], Liang Wang[1*]

[1]Department of Hematology, Beijing Tongren Hospital, Capital Medical University, No. 1 Dongjiaominxiang, Dongcheng District, Beijing 100730, China.

[*]Correspondence to: Liang Wang, Email: wangliangtrhos@126.com

[†]*Li-jie Zeng and Wei-xiang Lu contributed equally.*

Li-jie Zeng, Email: zenglijie1231@163.com

Wei-xiang Lu, Email: luweixiang0727@163.com

Yuan-zheng Liang, Email: yuanzheng_liang@163.com

Jing Yang, Email: yangj_08@163.com

Jin-yan Zhu, Email: zhujinyan1025@126.com

Main text word count:

3,397 words, excluding abstract, methods, references, tables, and figure legends.

Figures and supplementary materials:

8 main figures, 7 supplementary figures, and 4 supplementary tables.

The total number of pages：12

Supplementary Figure1

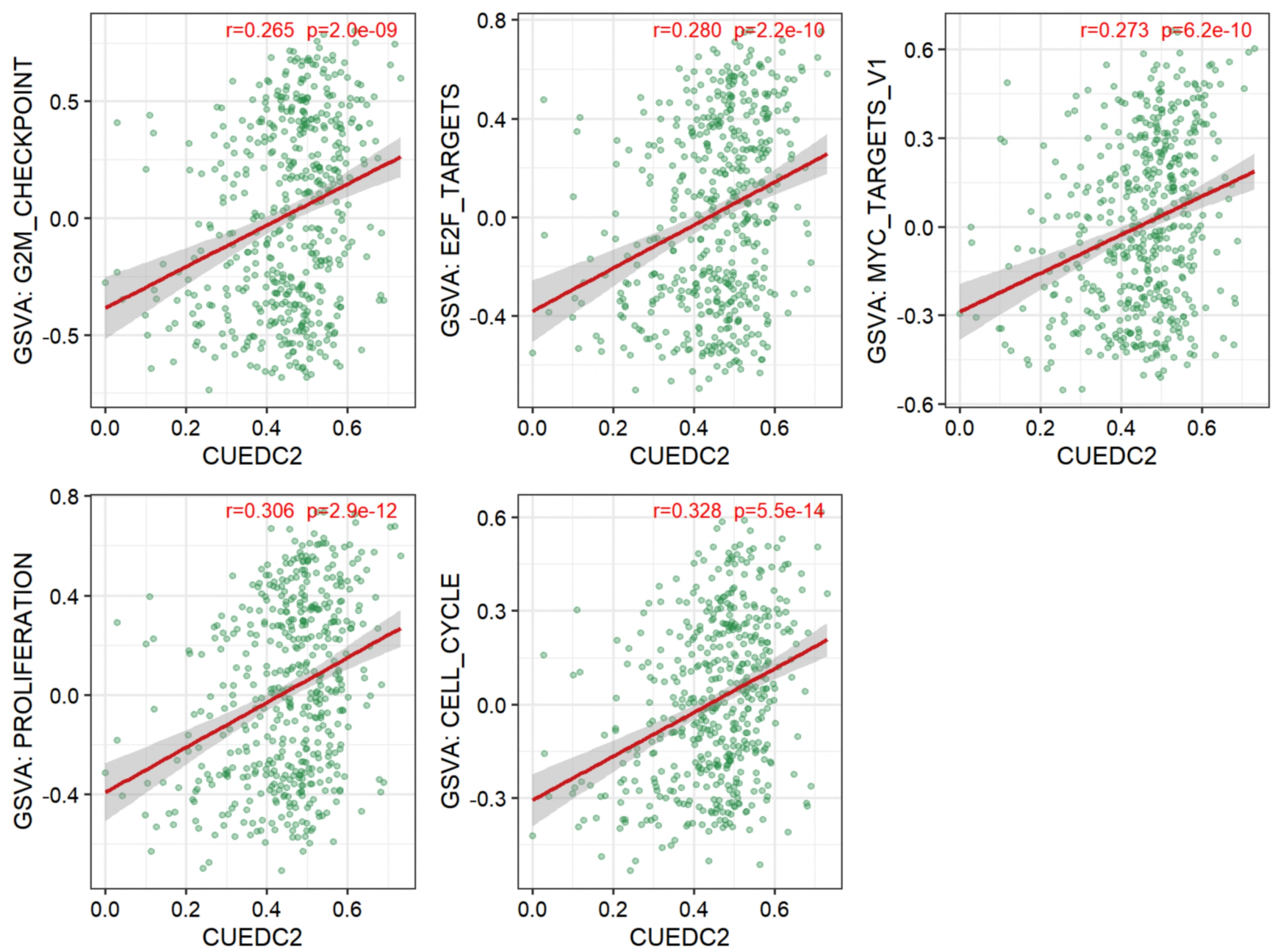

Fig. S1 CUEDC2 expression correlates with proliferation-related gene-set scores in DLBCL. Correlations of CUEDC2 expression with GSVA scores for G2M_CHECKPOINT, E2F_TARGETS, MYC_TARGETS_V1, PROLIFERATION, and CELL_CYCLE. Green points represent individual samples; red lines show fitted trends, with gray uncertainty bands. Correlation coefficients and P values are displayed in the panels.

Supplementary Figure2

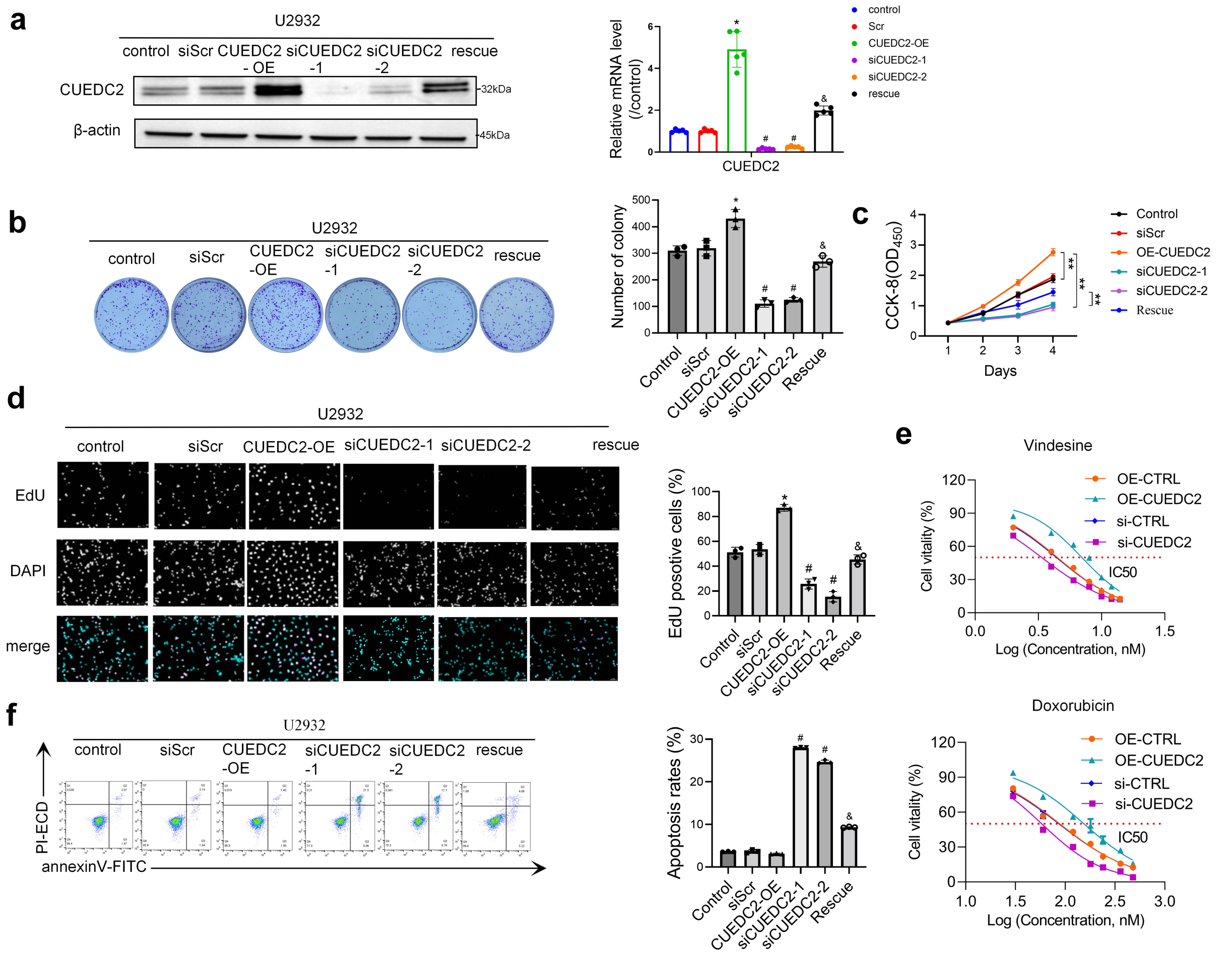

Fig. S2 CUEDC2 supports proliferation and chemoresistance in U2932 cells. (a) Western blot and qRT-PCR analyses confirming CUEDC2 overexpression, siRNA-mediated knockdown, and rescue. (b) Soft-agar colony formation and quantification of colony number. (c) CCK-8 assay of the indicated groups, initially seeded at 2,000 cells per group. (d) EdU incorporation and quantification of EdU-positive cells. Individual channels are shown in grayscale; merged images show white EdU signals and cyan DAPI-stained nuclei. (e) Vindesine (upper panels) and doxorubicin (lower panels) dose – response curves following CUEDC2 knockdown or overexpression; dashed red lines indicate 50% viability. (f) Annexin V/propidium iodide flow cytometry and quantification of apoptosis. Quantitative data are presented as mean ± SD from triplicate experiments. siScr, scrambled siRNA; CUEDC2-OE, CUEDC2 overexpression. *P < 0.05; **P < 0.01; ***P < 0.001, one-way ANOVA. #P < 0.05 versus control; &P < 0.05 versus the siCUEDC2 group;

these pairwise comparisons were assessed using t tests.

Supplementary Figure3

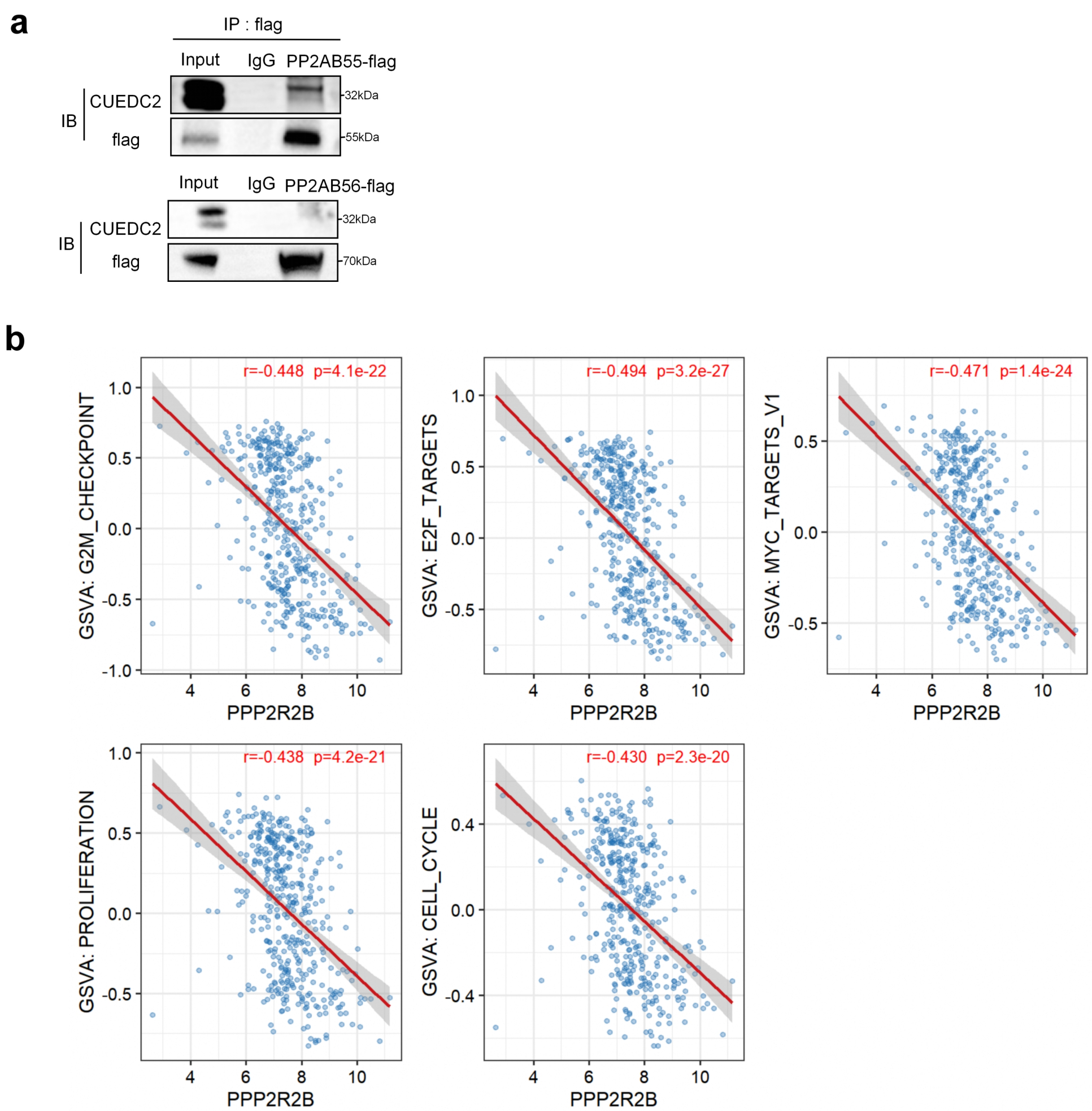

Fig. S3 PP2A-B55 associates with CUEDC2, and PP2A-B55β expression correlates with proliferation-related gene-set scores. (a) Reverse Co-IP using FLAG-tagged PP2A-B55 or PP2A-B56, followed by immunoblotting for CUEDC2. (b) Correlations of PPP2R2B (encoding PP2A-B55β) expression with GSVA scores for G2M_CHECKPOINT, E2F_TARGETS, MYC_TARGETS_V1, PROLIFERATION, and CELL_CYCLE. Blue points represent individual samples; red lines show fitted trends, with gray uncertainty bands. Correlation coefficients and P values are displayed in the panels.

Supplementary Figure4

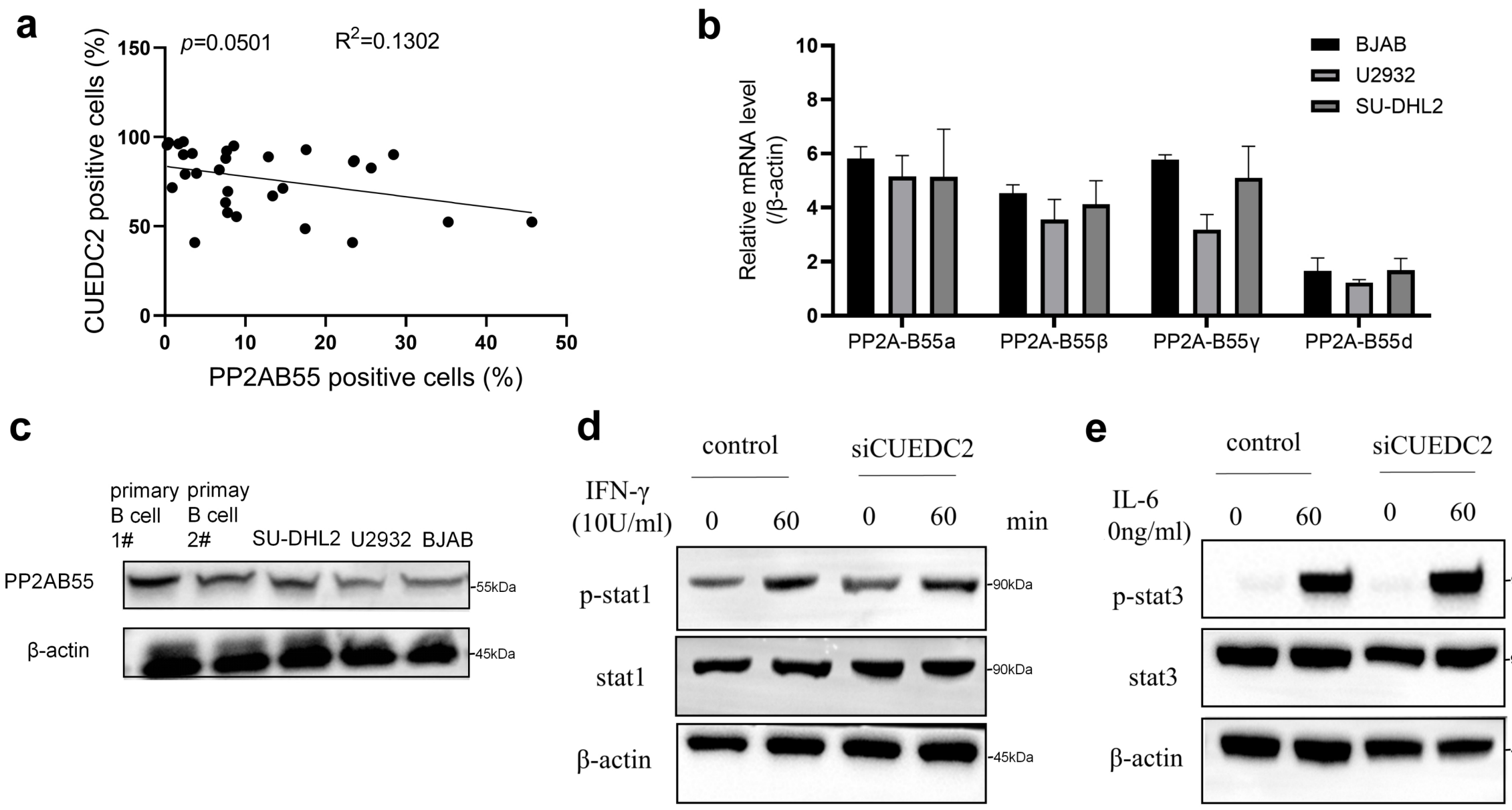

Fig. S4 Additional analyses of PP2A-B55 expression and cytokine signaling. (a) Relationship between CUEDC2-positive and PP2A-B55-positive cell percentages in clinical DLBCL samples; the fitted inverse trend has P = 0.0501 and R^2 = 0.1302. (b) Relative mRNA levels of B55α, B55β, B55γ, and B55δ in BJAB, U2932, and SU-DHL2 cells. Data are presented as mean ± SD (n = 3). (c) PP2A-B55 protein abundance in primary B cells from two donors and in DLBCL cell lines. (d) STAT1 phosphorylation before and 60 min after IFN-γ stimulation (10 U/mL) in control and CUEDC2-knockdown DLBCL cells. (e) STAT3 phosphorylation before and 60 min after IL-6 stimulation (10 ng/mL) in the corresponding groups.

Supplementary Figure5

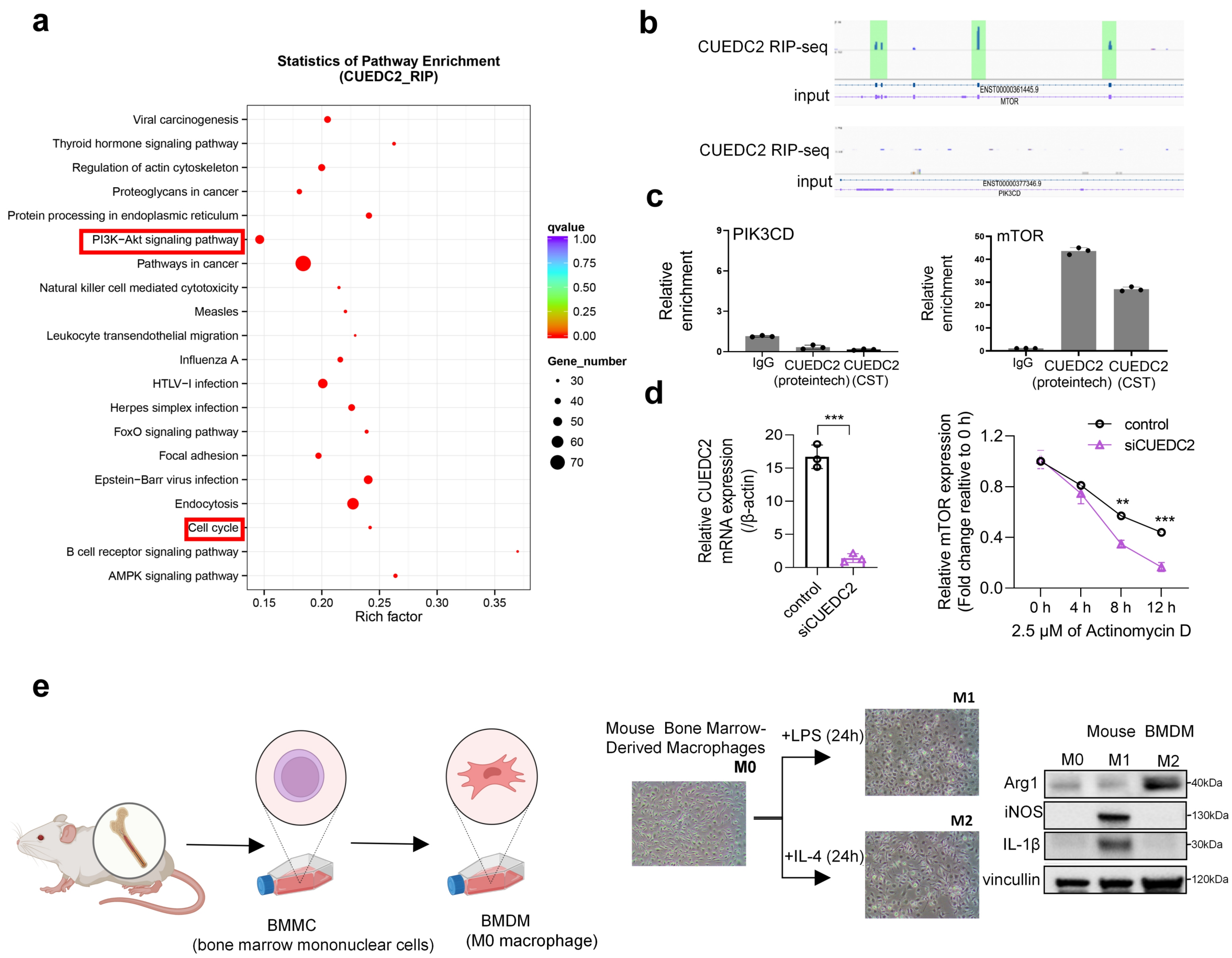

Fig. S5 CUEDC2-associated RNAs, mTOR mRNA stability, and BMDM preparation. (a) KEGG enrichment analysis of RNAs identified by CUEDC2 RIP-seq in SU-DHL2 cells. Bubble size indicates gene count and the color scale indicates the q value; red boxes highlight PI3K/AKT signaling and cell-cycle pathways. (b) Genome-browser tracks over mTOR and the PIK3CD background locus; highlighted regions indicate enriched signal. (c) RIP-qPCR analysis of PIK3CD (left) and mTOR (right) mRNA enrichment using CUEDC2 antibodies from Proteintech and Cell Signaling Technology, with IgG controls. (d) CUEDC2-knockdown validation and mTOR transcript decay after treatment with actinomycin D (2.5 μM) for 0, 4, 8, or 12 h. (e) Diagram of BMDM generation from bone marrow mononuclear cells, representative morphology, and immunoblotting for Arg1, iNOS, IL-1β, and vinculin. M0 denotes unpolarized macrophages; LPS and IL-4 treatments for 24 h indicate M1 and M2 polarization

conditions, respectively. Data in (c) and (d) are presented as mean ± SD (n = 3). In (d), siCUEDC2 is compared with control; **P < 0.01; ***P < 0.001. Schematic in (e): Created in BioRender. Zeng, L. (2026) https://BioRender.com/eom30ol (publication license agreement no. CR2A95N6QH).

Supplementary Figure6

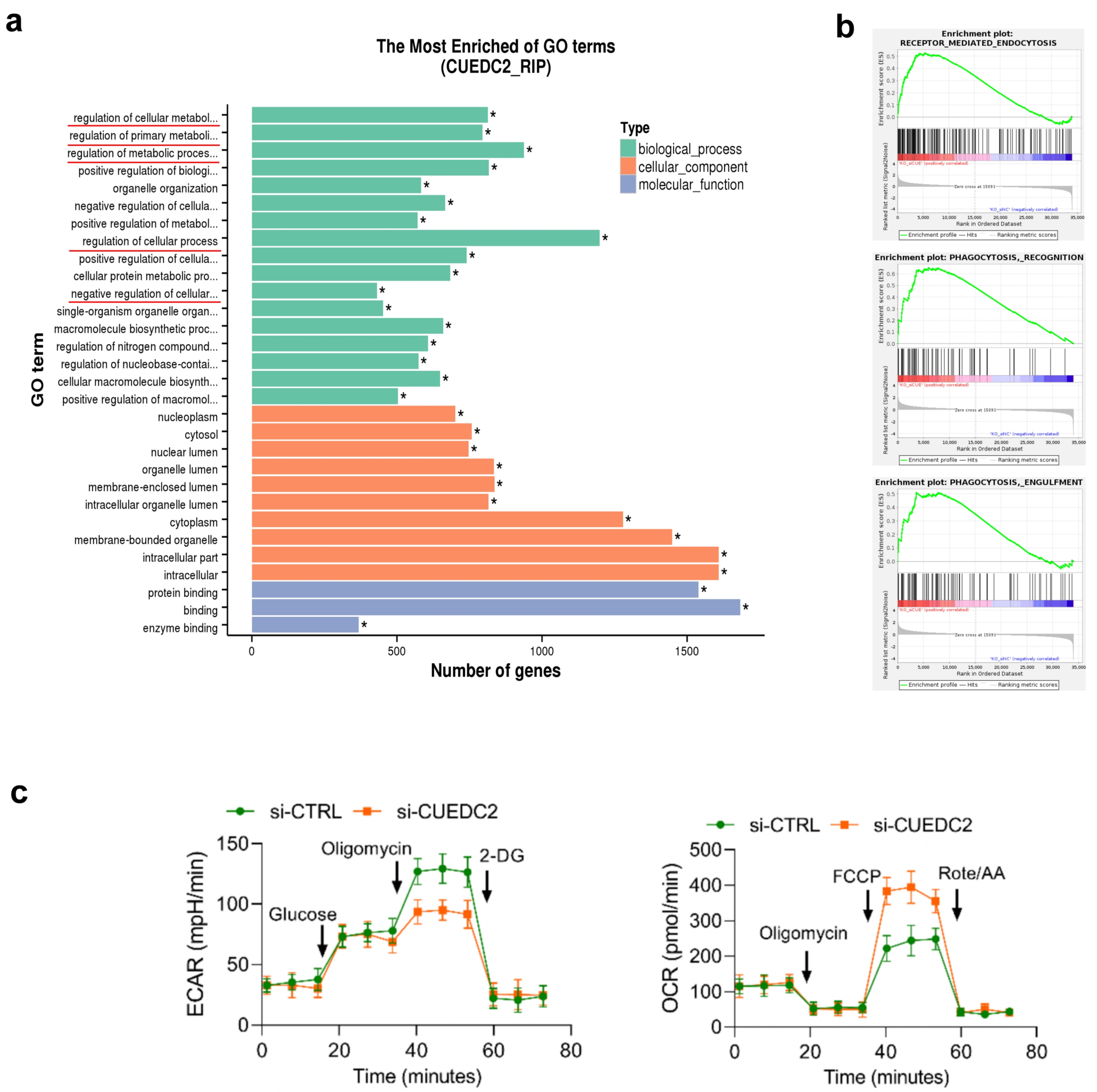

Fig. S6 Exploratory analyses of CUEDC2-associated macrophage functions and metabolism. (a) GO enrichment analysis of CUEDC2-associated RNAs in BMDMs. Green, orange, and blue-gray indicate biological process, cellular component, and molecular function categories, respectively. Enrichment analysis was performed using the GOseq R package with correction for gene-length bias; *corrected P < 0.05. Red underlines highlight metabolism-related terms. (b) GSEA plots for

receptor-mediated endocytosis, phagocytosis recognition, and engulfment. (c) Extracellular acidification rate (ECAR) after sequential addition of glucose, oligomycin, and 2-DG, and oxygen consumption rate (OCR) after oligomycin, FCCP, and rotenone/antimycin A. CUEDC2-depleted cells showed reduced ECAR and a greater FCCP-stimulated OCR response. Arrows indicate reagent additions. Green circles and orange squares indicate siCTRL and siCUEDC2, respectively. Data are presented as mean ± SD (n = 3).

Supplementary Figure7

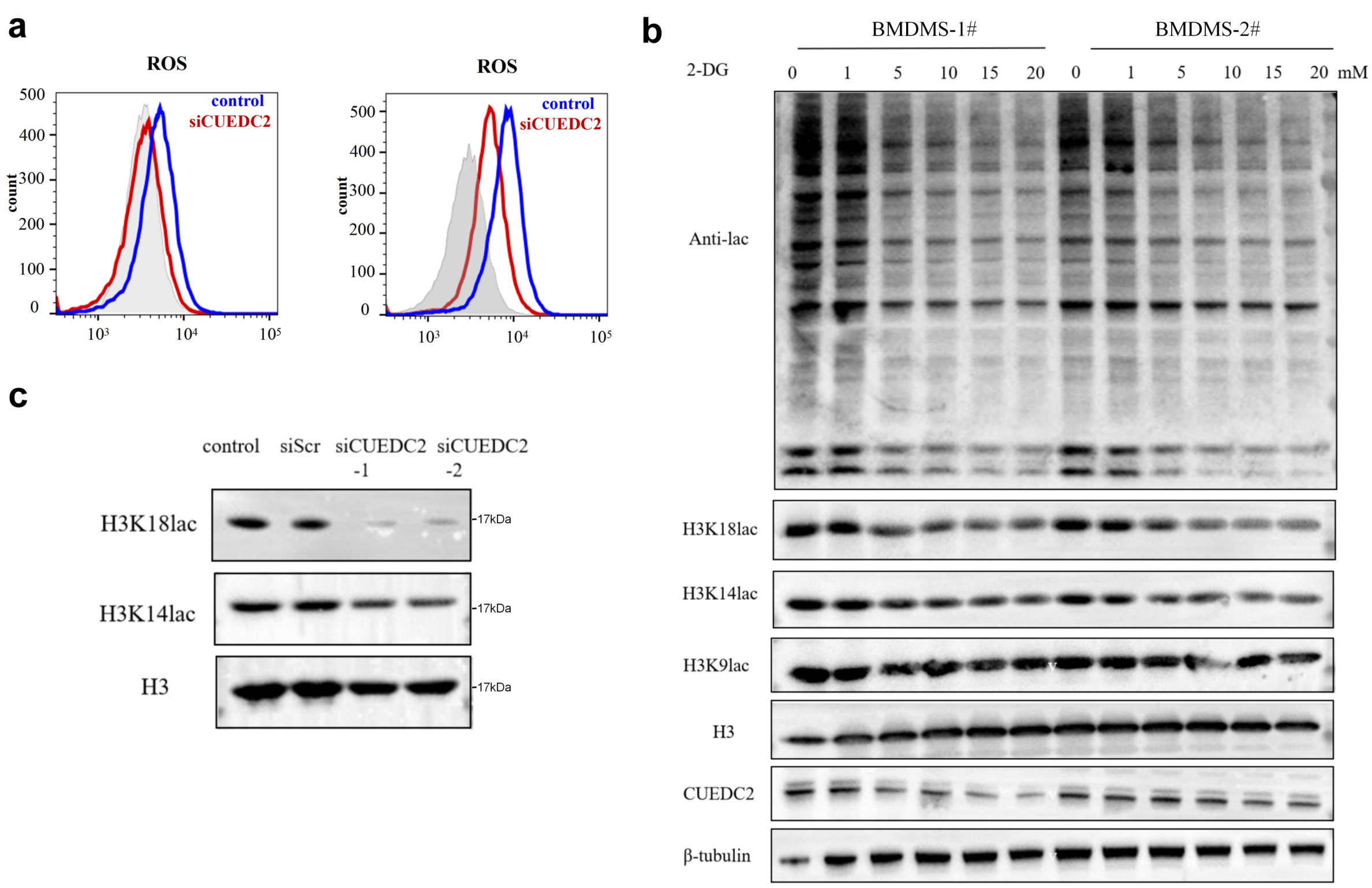

Fig. S7 CUEDC2 depletion, glycolysis inhibition, and macrophage lactylation. (a) Flow cytometry profiles of reactive oxygen species (ROS) signals in control (blue) and CUEDC2-knockdown (red) macrophages; the gray-shaded curve represents the isotype antibody control (background fluorescence). (b) Immunoblotting in two BMDM preparations treated with 0, 1, 5, 10, 15, or 20 mM 2-DG, showing pan-protein lactylation, H3K18 lactylation, H3K14 lactylation, H3K9

lactylation, total H3, CUEDC2, and β-tubulin. (c) H3K18 and H3K14 lactylation and total H3 in control, scrambled-siRNA, siCUEDC2-1, and siCUEDC2-2 BMDMs. Panels (b) and (c) represent distinct perturbations: pharmacological glycolysis inhibition and CUEDC2 knockdown, respectively.

Supplementary Table 1 Clinical Characteristics of patients

Characteristics	All patients (n=30)
Age(years), n (%)	
>60	13（43%）
≤60	17（57%）
Sex, n (%)	
Male	18（60%）
Female	12（40%）
Ann Arbor stage， n (%)	
I-II	14（47%）
III-IV	16（53%）
IPI score, n (%)	
0-2	10（33%）
3-5	20（67%）
P53 abnormality*, n (%)	4（13%）
DEL or DHL, n (%)	10（33%）
Cell of origin, n (%)	
GCB	18（60%）
non-GCB	12（40%）

** TP53 alteration was defined as either: (i) strong nuclear immunohistochemical expression (>30% positive tumor cells) indicating TP53 mutation, or (ii) complete loss of TP53 expression (0%), or (iii) detection of pathogenic TP53 mutation by next-generation sequencing.*

Supplementary Table 2 Reagents

antibody	catalog	source
CUEDC2	12294	Cell Signaling Technology
PPP2R2A/B/C	67783-1-lg	proteintech
PPP2R5D	5687	Cell Signaling Technology
β-actin	4970	Cell Signaling Technology
mTOR	2972	Cell Signaling Technology
Phospho-mTOR (Ser2448)	2971	Cell Signaling Technology
IκBα	4812	Cell Signaling Technology
Phospho-IκBα(Ser32/36)	9246	Cell Signaling Technology
p65	8242	Cell Signaling Technology
LaminB1	13435	Cell Signaling Technology
iNOS	2982	Cell Signaling Technology
Arg1	93668	Cell Signaling Technology
vincullin	13901	Cell Signaling Technology
stat1	9172	Cell Signaling Technology
Phospho-Stat1 (Tyr701)	9167	Cell Signaling Technology
stat3	9139	Cell Signaling Technology
Phospho-Stat3 (Tyr705)	9145	Cell Signaling Technology
IL-1β	12242	Cell Signaling Technology
H3	9715	Cell Signaling Technology
H3K14lac	PTM-1414RM	PTM BIO
H3K18lac	PTM-1406RM	PTM BIO
H3K9lac	PTM-1419RM	PTM BIO
Anti-lac	PTM-1401	PTM BIO

Supplementary Table 3 Primers

Gene name	Former primer (3'to5')	Reverse primer (5'to3')
CUEDC2	GCATCATGTCCCCTATTGTGC	CTGGAGAGGATCGTCAGTGC
PPP2R2B	TGTATTCACCCCTACGATGAACC	CCACACGGGAGAATTACTAGCG
PPP2R2A	ACAGTTCTGAAGCCTCGCAA	GCCAGGCTGTGTGAAGGATT
PPP2R2C	GTTGGTGGACAGGAGTGAGT	CCGGGTCGTCATCTTCCAG
β-actin	CCACGAAACTACCTTCAACTCC	GTGATCTCCTTCTGCATCCTGT
mTOR	TCTTGACTCATCTCTCGGAGTT	ATGCTTGGAACCGGACCTG
PIK3CD	GAAGAGCGGCTCATACTGGG	AAGGAGGAGAATCAGAGCGTT

Supplementary Table 4 siRNAs

Gene name	sense (5'to3')	antisense (5'to3')
NC	UUCUCCGAACGUGUCACGUTT	ACGUGACACGUUCGGAGAATT
mouse GAPDH-420	CACUCAAGAUUGUCAGCAATT	UUGCUGACAAUCUUGAGUGAG
Cuedc2-mus-1	AGCUGAAAAGCUCGAAGAATT	UUCUUCGAGCUUUUCAGCUTT
Cuedc2-mus-2	GACCAUCAGAGGAGAACUUTT	AAGUUCUCCUCUGAUGGUCTT
CUEDC2-Homo-1	GAGCGAUGCCAGGAACAAATT	UUUGUUCCUGGCAUCGCUCTT
CUEDC2-Homo-2	GCCCGAAAUGCUCAAAGAATT	UUCUUUGAGCAUUUCGGGCTT
PPP2R2A-Homo-1	GGUCGAUAUAUGAUGACUATT	UAGUCAUCAUAUAUCGACCTT
PPP2R2A-Homo-2	GCAGCAGAAUUUCAUCCAATT	UUGGAUGAAAUUCUGCUGCTT
